# Tumor-Derived Exosomal Fatty Acids Reprogram Neutrophils to Drive Neutrophil Extracellular Traps Formation and Lung Cancer Progression

**DOI:** 10.34133/research.1278

**Published:** 2026-05-11

**Authors:** Lulu Han, Yuxin Chen, Ruchen Wu, Junze Chen, Zhaokai Chen, Ying Cai, Shuxin Huang, Haoqing Gu, Xiaoyi Zhuang, Yanfang Lv, Huizhong Li, Liantao Li, Gang Wang

**Affiliations:** ^1^ Cancer Institute, Xuzhou Medical University, 209 Tongshan Road, Xuzhou 221004, China.; ^2^ Center of Clinical Oncology, The Affiliated Hospital of Xuzhou Medical University, 99 West Huaihai Road, Xuzhou 221002, China.; ^3^ Jiangsu Center for the Collaboration and Innovation of Cancer Biotherapy, Xuzhou Medical University, 209 Tongshan Road, Xuzhou 221004, China.

## Abstract

Tumor-infiltrating neutrophils are increasingly recognized as key drivers of cancer progression, but the mechanisms that govern their protumoral reprogramming remain elusive. Here, we demonstrated that lung cancer-derived exosomes (LDEs) deliver free fatty acids into neutrophils, triggering lipid accumulation, fatty acid β-oxidation, and mitochondrial reactive oxygen species production. This metabolic reprogramming culminates in enhanced neutrophil extracellular traps (NETs) formation, which accelerates lung cancer growth. Mechanistically, Ras-related protein Rab-34 (Rab34), a small guanosine triphosphatase, regulates neutrophil uptake of LDEs. Knockdown of Rab34 in neutrophils dramatically attenuated LDEs-induced lipid accumulation, fatty acid β-oxidation activation, and NETs formation, thereby mitigating neutrophil-involved lung cancer progression in vivo. Deoxyribonuclease I-mediated NETs degradation further confirmed the dependency of lung cancer growth on NETs. These findings uncovered a Rab34-dependent way by which LDEs reprogram neutrophils via exosomal free fatty acids, offering Rab34 and NET-associated pathways as potential therapeutic targets in lung cancer.

## Introduction

Lung cancer continues to be the primary cause of cancer-related mortality worldwide [1]. Although therapeutic approaches have advanced, their efficacy remains limited by the complex tumor microenvironment (TME), which facilitates tumor growth and angiogenesis while negatively modulating antitumor immunity [2]. Neutrophils constitute the predominant immune cell subpopulation in lung cancer TME [3,4]. Strikingly, numerous publications have shown that neutrophil depletion abrogates lung cancer growth in mice [5–7]. We and others have reported that tumor-infiltrating neutrophils mount a convergent reprogramming into a protumoral state, which fuels tumor progression [8–10]. However, the mechanisms driving this protumoral reprogramming are poorly defined.

Tumor-infiltrating neutrophils accelerate lung cancer progression by forming neutrophil extracellular traps (NETs) [11,12]. These web-like structures consist of DNA, myeloperoxidase (MPO), histones, and antimicrobial proteins [13], and they play a crucial role in combating pathogens. There is, however, growing consensus that NETs play a key role in facilitating cancer progression [14,15]. Inhibiting NETs formation or targeting their components is being explored as a potential therapeutic strategy in lung cancer treatment [11]. Elevated NETs level in cancer patients is correlated with metastasis and therapy resistance [16,17]. NETs triggered by tumor cells combines with integrin αvβ1 and matrix metalloproteinase-9 (MMP-9) to jointly activate transforming growth factor-β, leading to epithelial–mesenchymal transition of tumor cells and low chemotherapy efficacy [18]. Recently, our study found that lung cancer cells induced neutrophil activation and release of NETs-associated proteins, such as MMP-9, exacerbating lung cancer growth [10]. However, the mechanisms by which lung cancer cells trigger NETs formation remain poorly understood.

Tumor cells release soluble factors including exosomes, proteins, and lipids to communicate with various immune cells [19–21]. As nanoscale extracellular vesicles, tumor-associated exosomes are pivotal mediators of intercellular communication, especially in the TME [22]. These vesicles consist of a lipid bilayer encapsulating proteins, RNA, and lipids, which enables them to transfer bioactive molecules to various immune cells [22]. The lipid composition of exosomes influences their biophysical properties, such as membrane fluidity and stability, which in turn affect their interactions with recipient cells. In the context of immunity, tumor-derived exosomes employ immunosuppressive lipids to modulate immune cell function [4,22]. This not only impedes dendritic cell maturation, T cell activation, and proper macrophage polarization but also fosters an immunosuppressive microenvironment that fuels tumor progression [23,24]. Thus, understanding the specific roles of exosome lipids in mediating immune responses, particularly in relation to NETs formation, is crucial for developing therapeutic strategies aimed to reactivate antitumor immunity in lung cancer.

Here, we show that free fatty acid (FFA)-carrying exosomes from lung cancer cells (LDEs) reprogram neutrophils toward a protumoral state, equipping them with the capacity to produce NETs and promote tumor growth. Uptake of LDEs resulted in increased intracellular fatty acids and subsequently fatty acid β-oxidation (FAO), mitochondrial reactive oxygen species (mtROS) production, and thus NETs formation. Furthermore, the small guanosine triphosphatase (GTPase) protein Rab34 was identified as a critical regulator of exosome uptake in neutrophils. Hence, our findings revealed a mechanistic insight into neutrophil-mediated tumor progression and provided Rab34 and NET-associated pathways as potential therapeutic targets.

## Results

### Exposure to lung cancer cells led to lipid accumulation of neutrophils

**Fig. 1.**
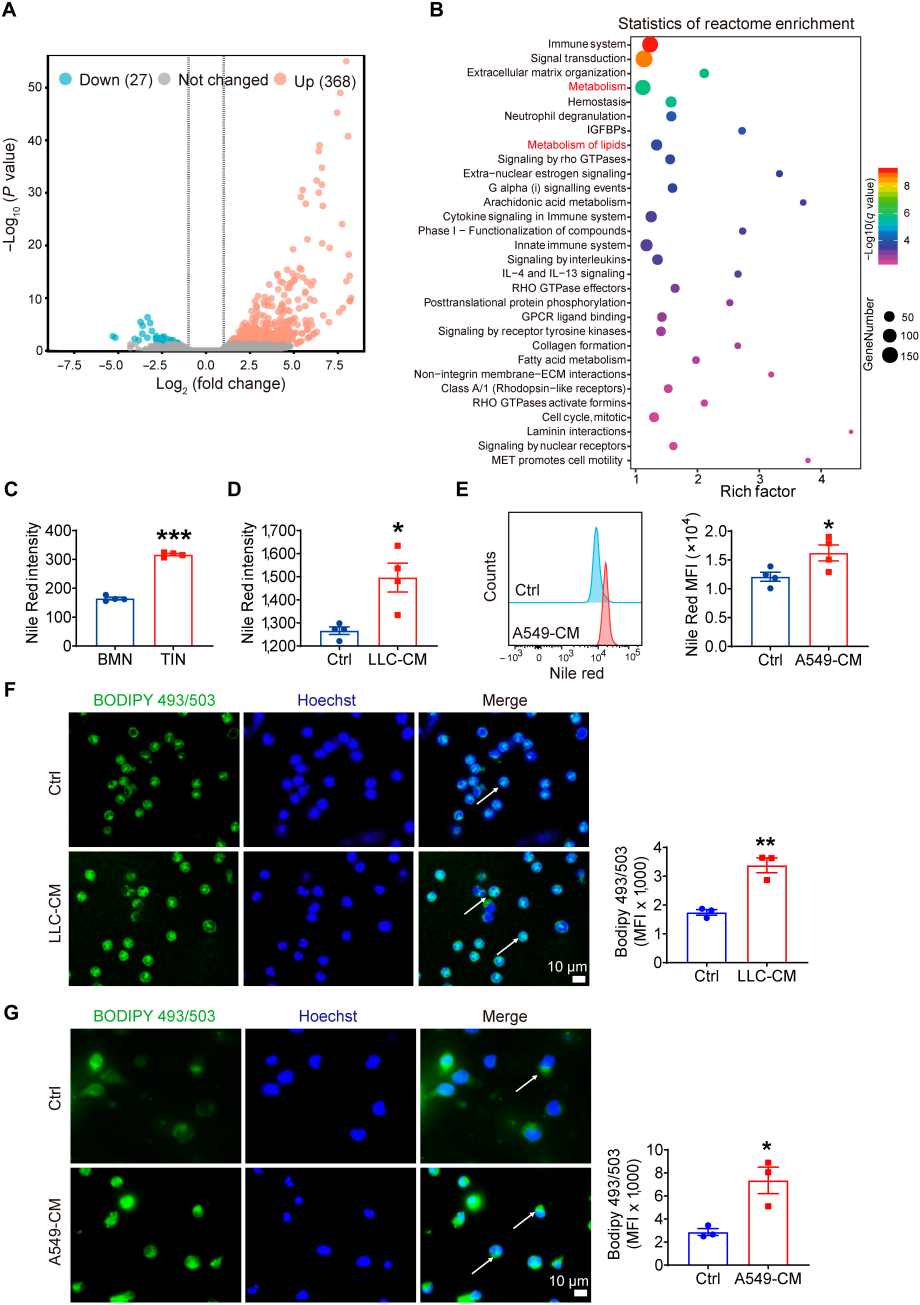
Exposure to lung cancer cells led to lipid accumulation of neutrophils. (A) Volcano plots showing differentially expressed genes following A549-conditioned medium (CM) stimulation. (B) Statistics of reactome enrichment analysis in A549-CM-treated neutrophils compared with control neutrophils. (C) Nile Red staining for mouse bone marrow neutrophils (BMNs) and tumor-infiltrated neutrophils (TINs). (D) BMNs were cultured with LLC-CM for 4 h. Lipid levels were measured by Nile Red staining with Cytation 3 Cell Reader. (E) Human differentiated HL-60 (dHL-60) neutrophils were cultured with A549-CM for 4 h. Lipid levels were measured by Nile Red staining with flow cytometry. (F and G) Lipid levels in BMNs (F) and dHL-60 cells (G) were measured by BODIPY 493/503 staining with a confocal microscope. All data are shown as means ± standard error of the mean (SEM) (*n* = 3 to 4, *P < 0.05, **P < 0.01, and ***P < 0.001). MFI, mean fluorescence intensity.

To investigate how lung cancer promotes neutrophil activation during tumor progression, we performed RNA sequencing (RNA-seq) of neutrophils exposed to lung cancer cell-conditioned medium (CM). Reactome enrichment analysis revealed that lung cancer-educated neutrophils up-regulated lipid metabolism-related genes, with a pronounced enrichment in lipid metabolic pathways compared to controls (Fig. 1A and B), suggesting enhanced lipid-handling capacity. Nile Red staining confirmed substantial lipid accumulation in tumor-infiltrating neutrophils sorted from mouse Lewis lung carcinoma (LLC) tumors, supporting these transcriptomic findings (Fig. 1C). To validate these observations, we incubated mouse bone marrow neutrophils (BMNs) and human neutrophil-like differentiated HL-60 (dHL-60) cells [25,26] with species-matched CM from either mouse (LLC) or human (A549) lung cancer cell lines. Nile Red staining intensity markedly increased in neutrophils cultured with lung cancer cell-CM compared to controls (Fig. 1D and E). In addition, neutrophils cultured with lung cancer cell-CM exhibit elevated lipid content, as clearly confirmed by BODIPY 493/503 fluorescence staining (Fig. 1F and G). These results demonstrated that lung cancer cells promoted lipid accumulation in neutrophils, suggesting that tumor-derived factors induce the formation of lipid-laden neutrophils both in vivo and in vitro*.*

### Lung cancer cell-derived exosomes potentiated lipid accumulation in neutrophils, thereby boosting their protumoral function

**Fig. 2.**
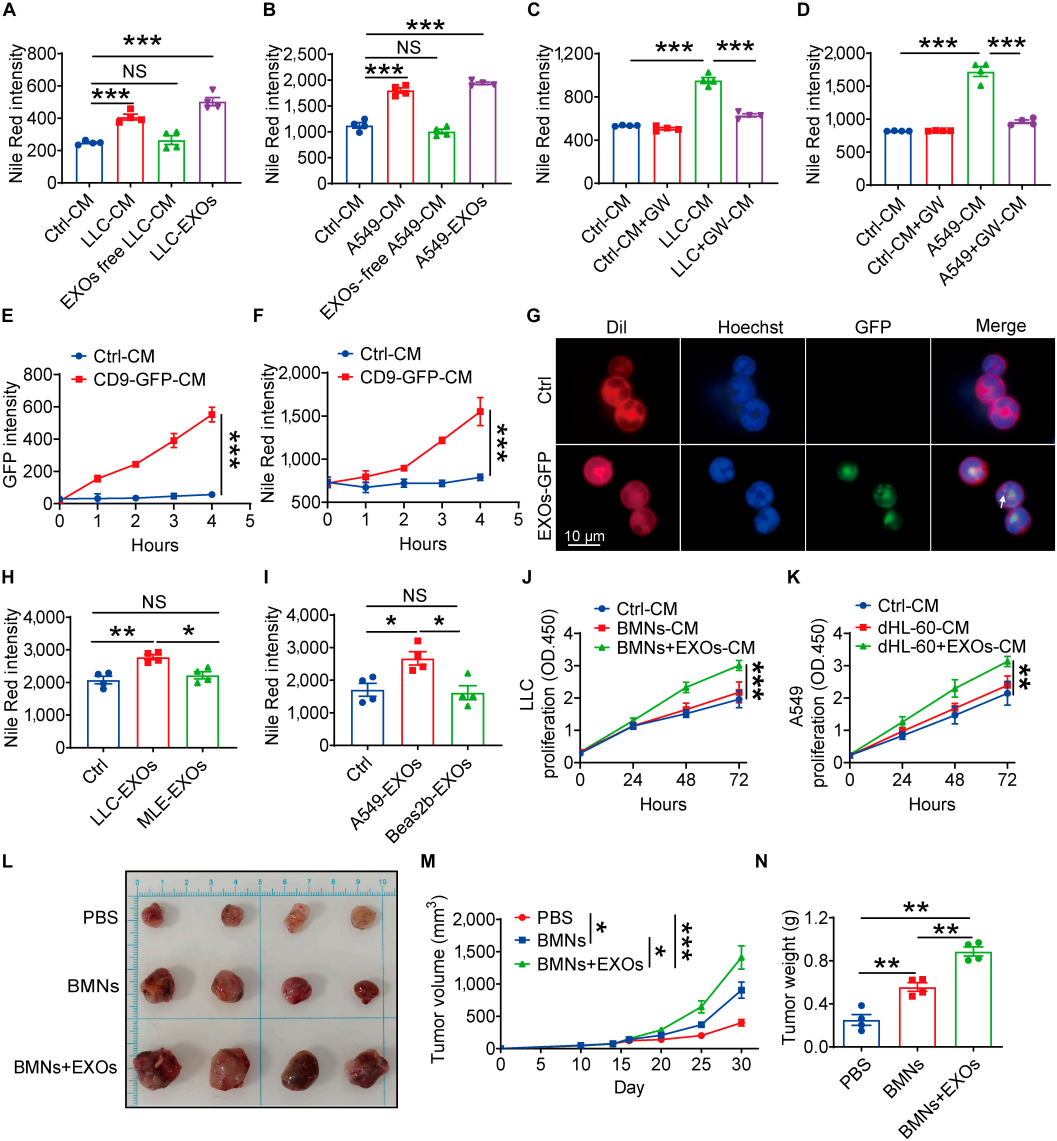
Lung cancer cell-derived exosomes potentiated lipid accumulation in neutrophils, thereby boosting their protumoral function. (A) Mouse bone marrow neutrophils (BMNs) were cultured with LLC-conditioned medium (CM), exosomes (EXOs)-free LLC-CM or LLC-EXOs for 4 h. Lipid levels were quantified using Nile Red staining with Cytation 3 Cell Reader. (B) Human differentiated HL-60 (dHL-60) neutrophils were cultured with A549-CM, EXOs-free A549-CM or A549-EXOs for 4 h. Lipid levels were quantified using Nile Red staining with Cytation 3 Cell Reader. (C) BMNs were cultured with LLC-CM or GW4869 (GW, 10 μM, 48 h)-treated LLC-CM. Lipid levels were quantified using Nile Red staining with Cytation 3 Cell Reader. (D) Human dHL-60 neutrophils were cultured A549-CM or GW-treated A549-CM. Lipid levels were quantified using Nile Red staining with Cytation 3 Cell Reader. (E and F) BMNs were cultured with CD9-GFP-CM or control-CM. (E) Green fluorescent protein (GFP) intensity of BMNs was detected by Cytation 3 Cell Reader at indicated time. (F) Lipid levels were quantified using Nile Red staining at indicated time. (G) LLC-GFP-exosomes taken up by BMNs were visualized via fluorescence microscopy. The arrow: GFP-exosome. (H) BMNs lipid levels were quantified using Nile Red staining after culturing with LLC-EXOs or murine lung epithelial cell line (MLE)-EXOs for 4 h. (I) dHL-60 cell lipid levels were measured by Nile Red staining after culturing with A549-EXOs or Beas2b-EXOs for 4 h. (J) The proliferation of LLC cells cultured in CM from BMNs (pretreated with or without LLC-exosomes) was determined by CCK-8 assay. (K) The proliferation of A549 cells cultured in CM from dHL-60 cells (pretreated with or without A549-exosomes) was determined by CCK-8 assay. (L) Tumor growth of LLC tumors receiving BMNs treated with or without exosomes. Representative tumor pictures are shown. (M) The tumor volumes were monitored. (N) Tumor weight analysis. All data are shown as means ± standard error of the mean (SEM) (*n* = 4, *P < 0.05, **P < 0.01, ***P < 0.001; NS, not significant). OD.450, optical density 450.

Tumor cells communicate with various immune cells by releasing soluble factors such as proteins, lipids, and exosomes [19,20]. To identify the key mediators of neutrophil lipid accumulation, we fractionated lung cancer cell-CM via ultracentrifugation to obtain precipitated exosomes and exosome-free soluble medium. The isolated exosomes were characterized by nanoparticle tracking analysis (typical size ~100 nm), positive for the exosomal marker CD9 and negative for calnexin by western blot, and displayed a cup-shaped morphology by transmission electron microscopy (Fig. S1A to C). Nile Red staining revealed that lung cancer cell-derived exosomes induced lipid accumulation in neutrophils instead of exosome-free soluble medium (Fig. 2A and B), indicating their central role in this process. Consistent with this mechanism, GW4869, an inhibitor of exosome biogenesis that blocks neutral sphingomyelinase (nSMase) activity (Fig. S1D), markedly reduced lipid accumulation in neutrophils (Fig. 2C and D). We also performed short hairpin RNA (shRNA)-mediated knockdown of nSMase (SMPD2) in A549 lung cancer cells (Fig. S1E). Knockdown of nSMase markedly reduced exosome secretion (Fig. S1F). CM from nSMase-knockdown cancer cells failed to induce lipid accumulation in neutrophils (Fig. S1G), mirroring the effect of GW4869 treatment. These findings directly demonstrated that lung cancer cell-derived exosomes critically regulate neutrophil lipid accumulation.

To further investigate whether the lipid accumulation of neutrophils was resulted from the uptake of lung cancer cell-derived exosomes, green fluorescent protein (GFP)-labeled exosomes were generated and collected with CD9-GFP-transduced LLC cells (Fig. S1H to J). Then, neutrophils were cultured with or without LLC-CD9-GFP CM. The results indicated a time-dependent increase in GFP and Nile Red levels in BMNs (Fig. 2E and F). Furthermore, confocal microscopy revealed intracellular punctate fluorescence, confirming active uptake of exosomes by neutrophils (Fig. 2G). Next, we isolated exosomes from murine lung epithelial (MLE) and Beas2b cells, representing noncancerous mouse and human lung epithelial cell lines. Neither MLE- nor Beas2b-derived exosomes increased neutrophil lipid levels, demonstrating that lung cancer cell-derived exosomes uniquely induce lipid accumulation in neutrophils (Fig. 2H and I). Collectively, these data demonstrated that lung cancer cell-derived exosomes induce lipid accumulation in neutrophils.

We next assessed whether LDEs promote tumor progression through neutrophil reprogramming. In vitro, LLC or A549 cells cultured with CM from LDE-treated neutrophils exhibited markedly increased viability compared to cells cultured with medium from untreated neutrophils (Fig. 2J and K). In vivo, adoptive transfer of LDE-primed neutrophils into tumor-bearing mice obviously enhanced tumor growth (Fig. 2L to N). Collectively, these data demonstrated that LDEs reprogram neutrophils toward a protumoral phenotype that directly supports lung cancer cell growth both in vitro and in vivo.

### Lung cancer cell-derived exosomes deliver FFAs into neutrophils to induce lipid accumulation

**Fig. 3.**
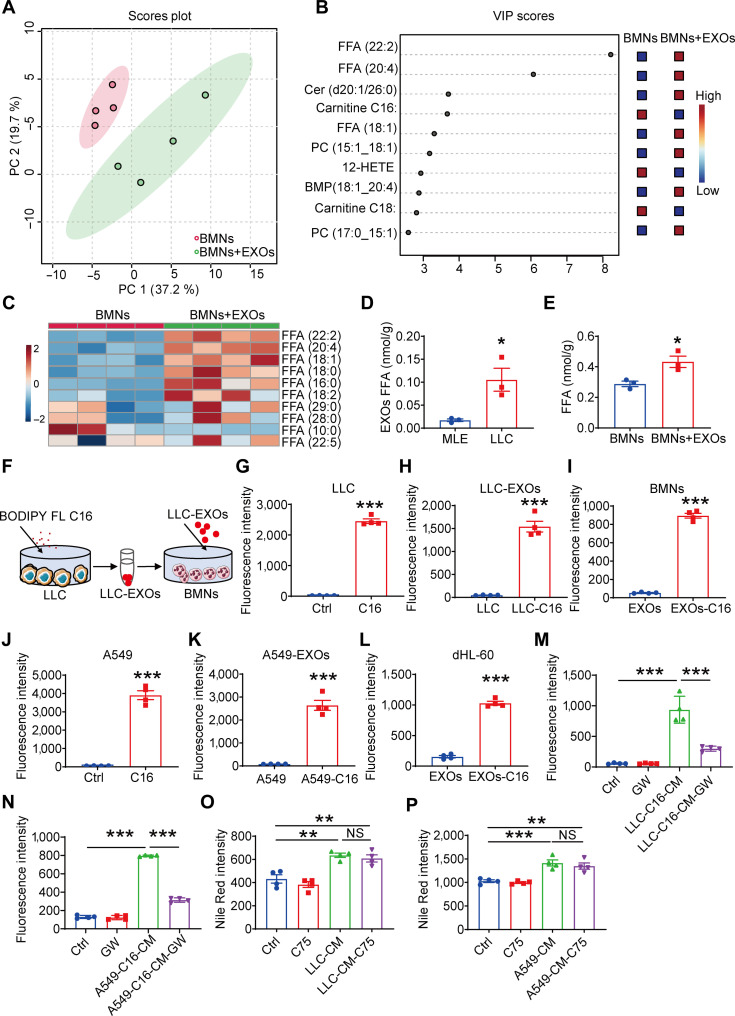
Lung cancer cell-derived exosomes deliver free fatty acids (FFA) into neutrophils to induce lipid accumulation. (A) PCA score plot and (B) variable importance in projection (VIP) plot of lipid metabolites from bone marrow neutrophils (BMNs) treated with or without LLC-EXOs. The VIP plot highlights the top 10 key metabolites contributing to group separation. (C) Heat map illustrating the fatty acids metabolic profiles. (D) FFA levels were measured in exosomes derived from murine lung epithelial (MLE) and LLC cells. (E) FFA levels were measured in BMNs cultured with exosomes. (F) Schematic diagram of fatty acid transfer experiment. (G) C16 fluorescence intensity was measured in LLC cultured with C16 for 24 h. (H) C16 fluorescence intensity was measured in exosomes from LLC. (I) C16 fluorescence intensity was measured in BMNs cultured with exosomes from LLC. (J) C16 fluorescence intensity was measured in A549 cultured with C16 for 24 h. (K) C16 fluorescence intensity was measured in exosomes from A549. (L) C16 fluorescence intensity was measured in differentiated HL-60 (dHL-60) cultured with exosomes from A549. (M) C16-LLC cells were cultured with or without GW4869. C16 fluorescence intensity was measured in BMNs cultured with LLC-conditioned medium (CM). (N) C16-A549 cells were cultured with or without GW4869. C16 fluorescence intensity was measured in dHL-60 cultured with LLC-CM. (O) BMNs were cultured with LLC-CM with or without C75 for 4 h. Lipid levels were quantified using Nile Red staining. (P) dHL-60 were cultured with A549-CM with or without C75 for 4 h. Lipid levels were quantified using Nile Red staining. All data are shown as means ± standard error of the mean (SEM) (*n* = 4, *P < 0.05, **P < 0.01, ***P < 0.001; NS, not significant).

To determine which lipids contribute to the lipid accumulation of neutrophils, lipid metabolites in neutrophils were analyzed using high-performance liquid chromatography-tandem mass spectrometry (HPLC-MS/MS) after culturing with or without lung cancer cell-derived exosomes. Partial least-squares discriminant analysis of lipidomics data from BMNs exposed to LLC-exosomes revealed distinct metabolic clustering compared to control neutrophils (Fig. 3A). Variable importance in projection analysis identified FFAs as the primary metabolites driving this separation (Fig. 3B). Lipid heatmap confirmed elevated FFA levels in exosome-stimulated neutrophils (Fig. 3C), suggesting that FFAs from lung cancer cell-derived exosomes may promote lipid accumulation. Comparative analysis demonstrated markedly higher FFA levels in exosomes from lung cancer cells than in those from noncancerous Beas2b and MLE cell lines (Fig. 3D and Fig. S2A). Neutrophils cultured with lung cancer cell-derived exosomes exhibited corresponding increases in lipid accumulation (Fig. 3E and Fig. S2B).

To monitor the intercellular transport of FFAs between lung cancer cells and neutrophils, a fluorescent fatty acid analog BODIPYTM FL C16 (C16) was used in lung cancer cell culture (Fig. 3F). Neutrophils showed markedly enhanced C16 fluorescence after exposure to exosomes from LLC or A549 cells preincubated with C16 for 4 h (Fig. 3G to L). GW4869-treated cancer cell-CM partially reduced this fluorescence accumulation (Fig. 3M and N), confirming direct fatty acid transfer via exosomes.

To directly test whether exosomal FFAs derived from cancer cell lipid metabolism are critical for neutrophil reprogramming, we generated stable fatty acid synthase-knockdown (FASN-KD) LLC and A549 cell lines using shRNA (Fig. S2C). Under serum-starved conditions, exosomes isolated from FASN-KD cells (LDEs-FASN-KD) exhibited markedly reduced FFA content compared to control exosomes (Fig. S2D and E). Accordingly, neutrophils lipid accumulation was markedly reduced after treatment with LDEs-FASN-KD (Fig. S2F and G). These results demonstrated that cancer cell FFA loading into exosomes is essential for exosome-induced neutrophil reprogramming.

To assess whether lung cancer cell factors stimulate neutrophil lipid accumulation through endogenous fatty acid synthesis, FASN was inhibited with the inhibitor C75. Treatment with C75 failed to attenuate lipid accumulation induced by cancer cell-CM (Fig. 3O and P), excluding de novo synthesis as a major contributor in neutrophils. Although tumor-educated neutrophils transcriptionally up-regulate lipid metabolism pathways, their actual lipid accumulation stems largely from exosome-delivered FFAs, not from de novo synthesis. Collectively, these findings demonstrated that lipid buildup in neutrophils results primarily from uptake of FFAs delivered by lung cancer cell-derived exosomes rather than self-synthesis.

### Lung cancer cell-derived exosomes promote neutrophil-induced tumor progression in a NETs-dependent manner

**Fig. 4.**
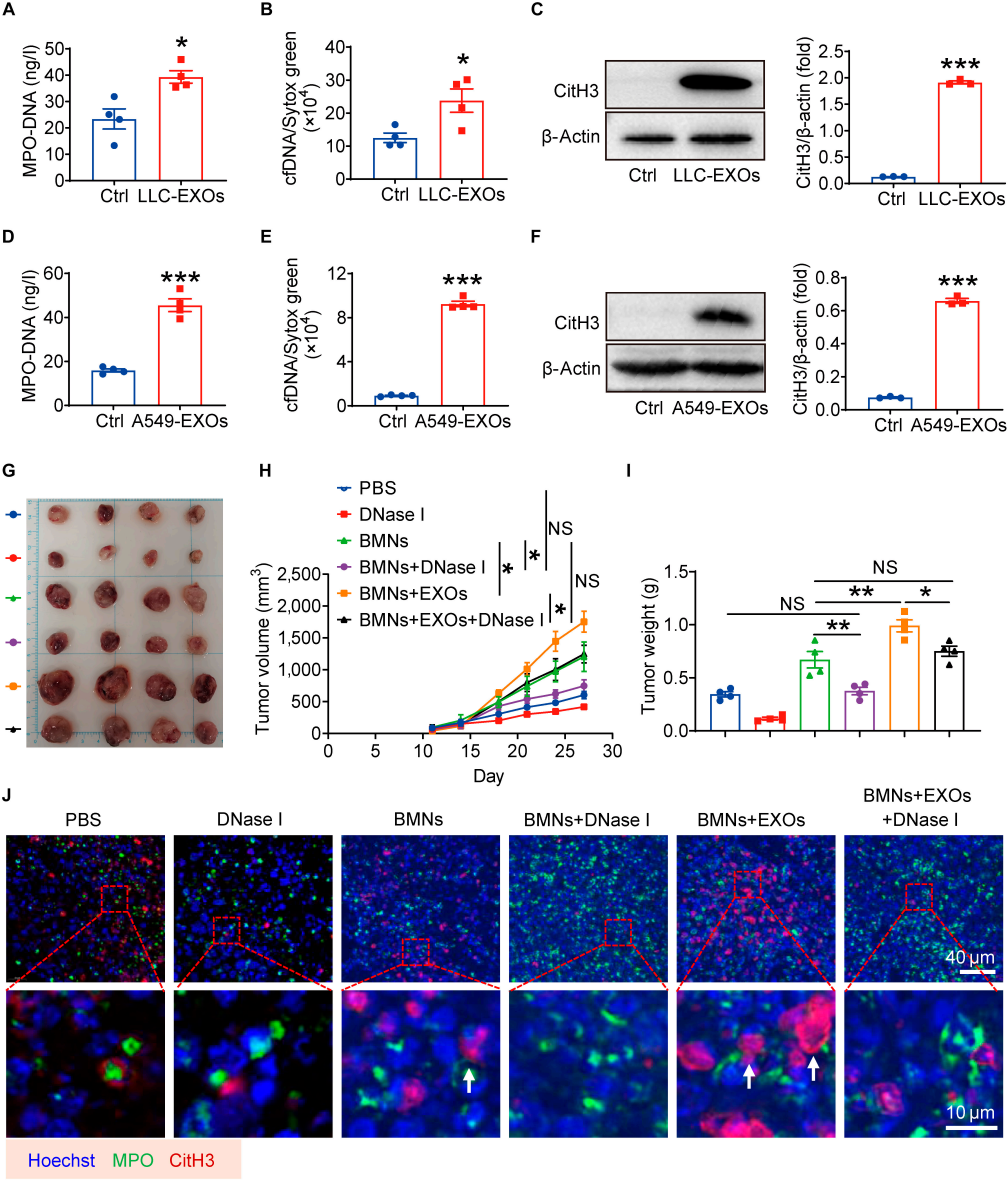
Lung cancer cell-derived exosomes promote neutrophil-induced tumor progression in a neutrophil extracellular traps (NETs)-dependent manner. (A) Mouse bone marrow neutrophils (BMNs) were cultured with LLC-EXOs for 4 h. Supernatant myeloperoxidase (MPO)-DNA levels were assessed by enzyme-linked immunosorbent assay (ELISA). (B) Cell-free DNA (cfDNA) levels in the supernatant were quantified using Sytox green. (C) Citrullinated histone H3 (CitH3) expressions were measured by western blot. (D) Human differentiated HL-60 (dHL-60) neutrophils were cultured with A549- EXOs for 4 h. Supernatant MPO–DNA levels were measured by ELISA. (E) Supernatant cfDNA levels were measured by Sytox green staining. (F) CitH3 expressions were measured by western blot. (G) LLC were injected subcutaneously with phosphate-buffered saline (PBS), BMNs, or BMNs and LLC-EXOs. Deoxyribonuclease I (DNase I) was injected intraperitoneally daily. Representative tumor pictures are shown. (H) The tumor volumes were monitored. (I) Tumor weights analysis. (J) Immunofluorescence staining for MPO and CitH3 in tumor tissues. All data are shown as means ± standard error of the mean (SEM) (*n* = 3 to 4, *P < 0.05, **P < 0.01, ***P < 0.001; NS, not significant).

Our prior study demonstrated that tumor-educated neutrophils promote lung cancer progression through MMP-9 synthesis and release [10]. Given that MMP-9 is also a key component of NETs [27], we hypothesized that LDEs may drive tumor progression by inducing NETs formation. To test this, we first examined whether LDEs affect MMP-9 expression. Indeed, LDE-treated neutrophils showed increased MMP-9 levels (Fig. S3A and B), suggesting that exosomes may simultaneously prime neutrophils for both MMP-9 release and NETosis. We next assessed NETs formation directly. Neutrophils stimulated with LDEs exhibited marked increases in classic NETs markers, including MPO–DNA complexes, cell-free DNA (cfDNA), and citrullinated histone H3 (CitH3), compared to controls (Fig. 4A to F). Immunofluorescence staining confirmed the presence of extracellular DNA fibers, characteristic of NET release, in LDE-treated neutrophils, while control neutrophils retained intact nuclei (Fig. S3C and D). Furthermore, treating neutrophils with increasing concentrations of LDEs (0 to 80 μg/ml) resulted in a graded increase in MPO–DNA, cfDNA, and CitH3 levels, accompanied by progressive lipid accumulation (Fig. S3E to H). These dose-response data further support a causal relationship between exosome exposure and NETs induction. These findings establish a direct role for lung cancer cell-derived exosomes in inducing NETs formation.

To assess the effect of NETs on lung cancer cells, we incubated the cells with CM from neutrophils that had been treated with or without LDEs and with or without deoxyribonuclease I (DNase I), an enzyme that degrades NETs. Cell Counting Kit-8 assays revealed that CM from LDE-primed neutrophils markedly enhanced lung cancer cell viability, an effect that was abolished by DNase I cotreatment (Fig. S3I and J), confirming that NETs directly promote lung cancer cell proliferation in vitro. To investigate whether exosome-induced NETs causally contribute to tumor progression, we therapeutically administered DNase I intraperitoneally to mice bearing LLC tumors. In line with the in vitro data, exosome-primed neutrophils markedly increased tumor growth, resulting in greater tumor volume and weight (Fig. 4G to I). Strikingly, DNase I-mediated degradation of NETs blocked the ability of exosome-educated neutrophils to promote tumorigenesis (Fig. 4G to I). Immunofluorescence analysis of tumor tissues further corroborated these observations. Tumors from mice that received exosome-primed neutrophils exhibited robust NET formation, as evidenced by the characteristic localization and morphology of MPO and CitH3 (Fig. 4J). The NETs induced by LDEs were effectively eliminated by in vivo DNase I treatment (Fig. 4J). These results demonstrated that NETs are essential for lung cancer cell-derived exosome-primed neutrophil-driven tumor progression.

### Lung cancer-derived exosomal fatty acids promote NETs formation through FAO pathways activation in neutrophils

**Fig. 5.**
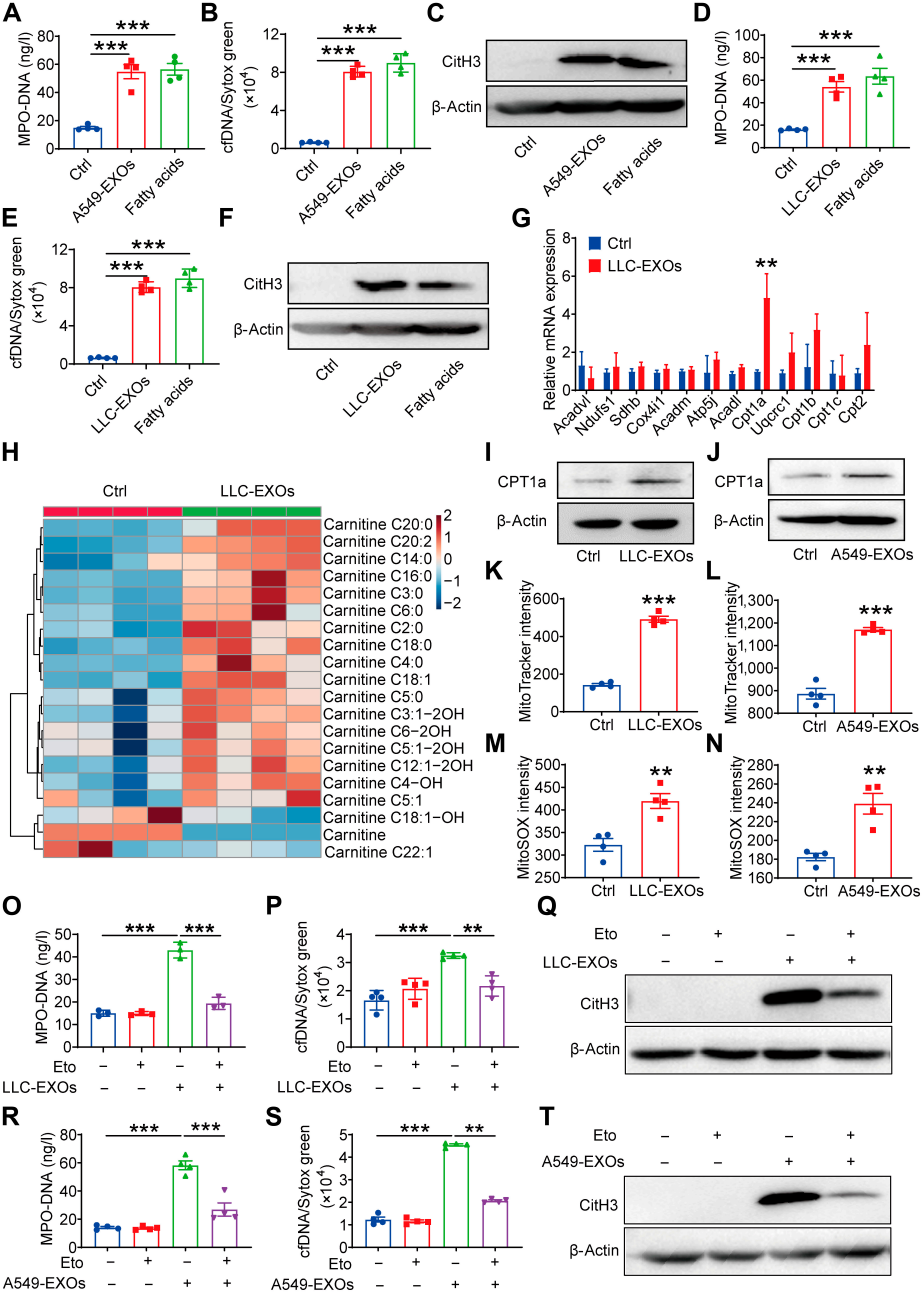
Lung cancer-derived exosomal fatty acids promote NETs formation through fatty acid β-oxidation (FAO) pathways activation in neutrophils. (A) Human differentiated HL-60 (dHL-60) neutrophils were cultured with A549-EXOs or free fatty acids for 4 h. Supernatant myeloperoxidase (MPO)-DNA levels were detected by enzyme-linked immunosorbent assay (ELISA). (B) Supernatant cell-free DNA (cfDNA) levels were measured by Sytox green staining. (C) Citrullinated histone H3 (CitH3) expressions were measured by western blot. (D) Mouse bone marrow neutrophils (BMNs) were cultured with LLC-EXOs or free fatty acids for 4 h. Supernatant MPO–DNA levels were measured by ELISA. (E) Supernatant cfDNA levels were measured by Sytox green staining. (F) CitH3 expressions were measured by western blot. (G) Gene expression analysis of FAO/oxidative phosphorylation pathways and (H) acylcarnitine heatmap in BMNs with or without LLC-EXOs treatment. (I and J) Analysis of CPT1a protein expression by western blot. (K) Quantification of mitochondrial mass in mouse BMNs cultured with LLC-EXOs, using MitoTracker staining and Cytation 3 Cell Reader. (L) Human dHL-60 neutrophils were cultured with A549-EXOs. Mitochondria mass was measured by MitoTracker staining with Cytation 3 Cell Reader. (M) Mouse BMNs were cultured with LLC-EXOs. mtROS was measured by mitoSOX staining with Cytation 3 Cell Reader. (N) Human dHL-60 neutrophils were cultured with A549-EXOs. mtROS was measured by mitoSOX staining. (O) Mouse BMNs were cultured with LLC-EXOs with 40 μM etomoxir (Eto) for 4 h. MPO–DNA levels were measured by ELISA. (P) cfDNA levels were measured by Sytox green staining. (Q) CitH3 expressions were measured by western blot. (R) dHL-60 neutrophils were cultured with A549-EXOs with 40 μM etomoxir for 4 h. MPO–DNA levels were measured by ELISA. (S) cfDNA levels were measured by Sytox green staining. (T) CitH3 protein expression was assessed by western blot. All data are shown as means ± standard error of the mean (SEM) (*n* = 3 to 4, **P < 0.01, ***P < 0.001).

To determine the contribution of exosomal fatty acids to NETs formation, we treated neutrophils with either LDEs or a combination of FFAs (palmitic, stearic, and oleic acid). Both treatments markedly induced NETs formation, as quantified by established NETs markers (Fig. 5A to F), demonstrating that exosome-derived fatty acids promote lipid accumulation in neutrophils, ultimately driving NETs formation. To determine whether specific fatty acid species differentially regulate NETosis, we treated neutrophils with individual FFAs, palmitic, stearic, or oleic acid. All 3 induced NETs formation and mtROS production comparable to the exosomes (Fig. S4A to D), demonstrating that both saturated and unsaturated FFAs drive NETosis. Crucially, the same FASN-KD LDEs failed to induce NETs formation. However, supplementing with exogenous FFAs completely restored their NETs-promoting effect (Fig. S5A to F). These results confirmed that the defective NETs induction stems specifically from the reduced FFA content in FASN-KD LDEs. To exclude a contribution from proteins, we heat-inactivated LDEs prior to neutrophil treatment. The heat-inactivated LDEs fully retained their ability to induce NETs formation (Fig. S5G to L), indicating that the NET-promoting activity is mediated by heat-stable components, primarily lipids, rather than protein cargo. Collectively, these findings establish that exosomal FFAs are both necessary and sufficient to drive NETs formation in neutrophils.

RNA-seq analysis revealed activation of FAO pathways in tumor-educated neutrophils (Fig. S5M). Subsequent quantitative real-time polymerase chain (PCR) analysis showed up-regulation of CPT1a, the rate-limiting enzyme for mitochondrial FAO, in exosome-treated neutrophils (Fig. 5G). To directly assess FAO flux, we measured the oxygen consumption rate (OCR) in BMNs treated with or without LDEs using a Seahorse analyzer. LDE treatment markedly increased both the basal and maximal OCRs. These increases were completely abolished by the CPT1a inhibitor etomoxir, confirming the specific activation of CPT1a-dependent FAO (Fig. S6A and B).

MS/MS analysis of neutrophil metabolites observed a marked remodeling of the carnitine pool, with reduced free carnitine and accumulation of long-chain species, including C16:0- and C18:1-carnitine (Fig. 5H), consistent with enhanced FAO flux. Western blot analysis confirmed CPT1a protein expression was increased following exosome treatment (Fig. 5I and J), indicating that exosomal fatty acids activate neutrophil FAO through CPT1a up-regulation.

Excessive FAO activation increases mitochondrial demand and mtROS production [28]. Consistent with this, MitoTracker and MitoSOX staining revealed elevated mitochondrial mass and mtROS levels in exosome-treated neutrophils (Fig. 5K to N). To determine whether FAO-driven mtROS contributes to NETs formation, CPT1a was inhibited with etomoxir during exosome exposure. Not surprising, etomoxir substantially suppressed exosome-induced NETs production (Fig. 5O to T). We further employed shRNA-mediated knockdown of CPT1a in HL-60-derived neutrophils. After confirming efficient CPT1a silencing at the protein level (Fig. S6C), we treated these cells with LDEs. The results aligned with our etomoxir data, demonstrating that CPT1a knockdown attenuated LDE-induced NETs formation (Fig. S6D to F). Similarly, the ROS scavenger N-acetylcysteine blocked NETs formation triggered by LDEs (Fig. S6G to L). These findings collectively indicate that FAO critically regulates NETs formation induced by exosomal fatty acids from lung cancer cells.

### Rab34 mediates neutrophil uptake of LDEs to promote NETs formation and tumor progression

**Fig. 6.**
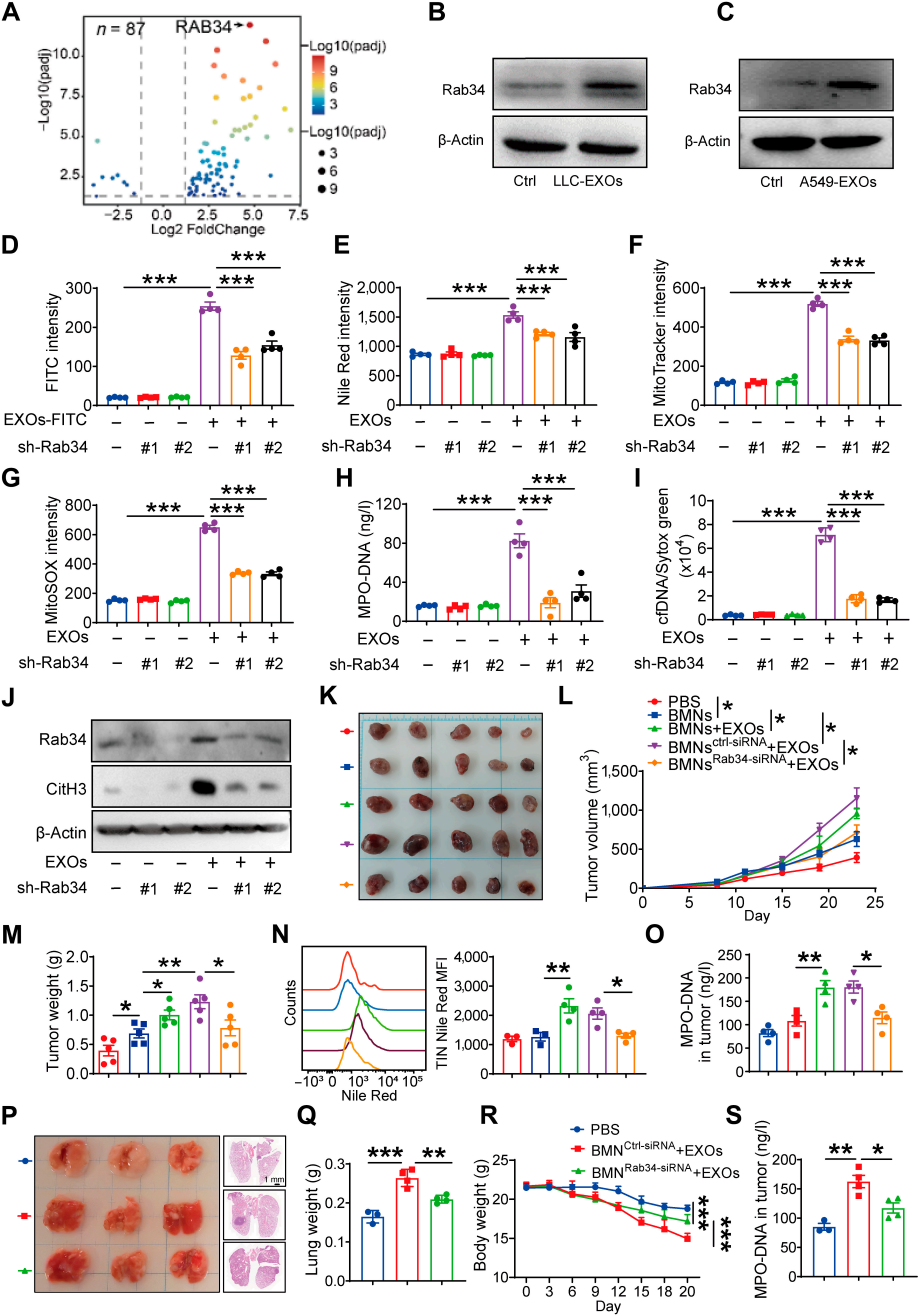
Rab34 mediates neutrophil uptake of lung cancer-derived exosomes to promote NETs formation and tumor progression. (A) Volcano plots showing differentially expressed genes associated lung the prognosis of cancer patients. (B) Rab34 expression in bone marrow neutrophils (BMNs) was analyzed by western blot following a 4-h incubation with LLC-EXOs. (C) Human differentiated HL-60 (dHL-60) neutrophils were cultured with A549-EXOs for 4 h. Rab34 expression was measured by western blot. (D and E) Human dHL-60 neutrophils with or without Rab34 knockdown were cultured with fluorescein isothiocyanate (FITC)-labeled EXOs for 4 h. (D) FITC intensity was detected by Cytation 3 Cell Reader. (E) Lipid levels were measured by Nile Red staining. (F) Human dHL-60 neutrophils with or without Rab34 knockdown were cultured with A549-exosomes for 4 h. Mitochondrial mass was detected by MitoTracker staining. (G) mtROS was detected by mitoSOX staining. (H) Myeloperoxidase (MPO)-DNA levels were measured by enzyme-linked immunosorbent assay (ELISA). (I) Cell-free DNA (cfDNA) levels were measured by Sytox green staining in supernatant. (J) Citrullinated histone H3 (CitH3) and Rab34 expressions were measured by western blot. (K) Tumor growth of LLC tumors receiving BMNs treated with or without Rab34-small interfering RNA (siRNA) liposomes. Representative tumor pictures are shown. (L) The tumor volumes were monitored. (M) Tumor weight analysis. (N) Tumor-infiltrating neutrophil lipid content was quantified by flow cytometry using Nile Red staining. (O) MPO–DNA levels in tumor tissues were assessed via ELISA. (P) Following orthotopic implantation of LLC cells into C57 mice, the animals received phosphate-buffered saline (PBS) or exosome-pretreated neutrophils 7 d later, with or without prior Rab34 knockdown. Representative macroscopic and histological images of hematoxylin and eosin (H&E)-stained lung tissue are presented. (Q) Lung weights and (R) body weights were recorded. (S) MPO–DNA levels in lung tissues were determined by ELISA. Data are presented as the means ± standard error of the mean (SEM) (*n* = 3 to 5, *P < 0.05, **P < 0.01, ***P < 0.001).

To identify key mediators of exosome internalization in neutrophils, we analyzed RNA-seq data and discovered that lung cancer cell-CM strongly up-regulated Rab34 (Fig. 6A), a small GTPase which reported in regulating vesicular trafficking [29–32]. Consistent with this, exosome treatment also markedly increased Rab34 expression in neutrophils (Fig. 6B and C). To determine whether Rab34 dominantly mediates the uptake of exosomes, shRNA-mediated knockdown of Rab34 was performed in HL-60-derived neutrophils (Fig. S7A). Rab34 deficiency substantially reduced both fluorescein isothiocyanate-exosome uptake and lipid accumulation (Fig. 6D and E), confirming its critical role in exosome internalization.

We next examined whether Rab34 mediates NETs formation upon exosome exposure. Rab34 knockdown prevented the exosome-induced increase in mitochondrial mass and mtROS (Fig. 6F and G) and markedly suppressed NETs formation (Fig. 6H to J). To determine whether Rab34 functions primarily in FFA delivery, we performed rescue and overexpression experiments. FFA supplementation fully restored NETs formation in Rab34-silenced neutrophils despite impaired exosome uptake (Fig. S7B to D), indicating that the downstream FAO–NETs axis remains intact when FFAs are supplied extracellularly. Conversely, Rab34 overexpression enhanced exosome uptake, lipid accumulation, and NETs formation (Fig. S7E to J). These results demonstrated that Rab34 is both necessary and sufficient for exosome uptake and subsequent neutrophil reprogramming.

However, the expression of NETs marker CitH3 was not affected by Rab34 depletion in lipopolysaccharide- or phorbol 12-myristate 13-acetate-stimulated neutrophils (Fig. S7K and L), suggesting that Rab34 specifically promotes NETs via regulating exosome uptake rather than neutrophil activation pathways.

To validate these findings in vivo, BMNs were pretreated with Rab34-small interfering RNA (siRNA) liposomes ex vivo and then injected into tumor-bearing mice, achieving selective Rab34 knockdown in tumor-infiltrating neutrophils but not macrophages (Fig. S7M and N). Using a murine lung cancer model coinjected with LLC cells and exosome-educated neutrophils, we demonstrated that Rab34 knockdown abrogated the tumor-promoting effects of these neutrophils (Fig. 6K to M). Furthermore, Rab34 knockdown markedly reduced lipid accumulation in tumor-infiltrating neutrophils and NETs formation in vivo (Fig. 6N and O). To validate our findings in a physiologically relevant setting, we established an orthotopic lung cancer model. Mice receiving control siRNA-treated BMNs exhibited obviously increased lung tumor burden, elevated intratumoral NETs formation, and more severe body weight loss. In contrast, Rab34 knockdown in neutrophils markedly attenuated all these parameters, confirming that Rab34-mediated exosome uptake drives lung cancer progression in the lung microenvironment (Fig. 6P to S). These findings definitively establish Rab34 as a critical mediator of exosome uptake in neutrophils and its essential role in promoting lung cancer progression.

### Exosomal FFAs are required for neutrophil-mediated tumor promotion in vivo

**Fig. 7.**
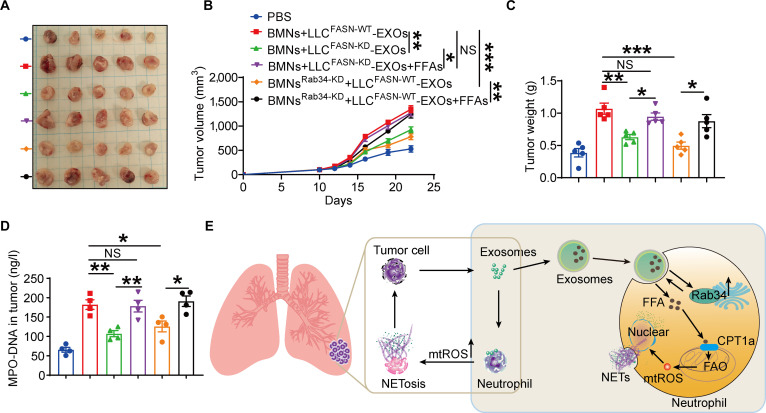
Exosomal free fatty acids (FFAs) are required for neutrophil-mediated tumor promotion in vivo. (A) Growth of LLC tumors in mice receiving bone marrow neutrophils (BMNs) pretreated with the indicated exosomes, with or without Rab34-small interfering RNA (siRNA) liposomes; representative images are shown. (B) Tumor volumes were monitored over time. (C) Final tumor weights were analyzed. (D) Myeloperoxidase (MPO)-DNA levels in tumor tissues were measured by enzyme-linked immunosorbent assay (ELISA). (E) Schematic illustrating how tumor-derived exosomal fatty acids reprogram neutrophils to drive NET formation and lung cancer progression. Data are presented as the means ± standard error of the mean (SEM) (*n* = 4 to 5, *P < 0.05, **P < 0.01, ***P < 0.001; NS, not significant).

To definitively establish that exosomal FFAs drive neutrophil-mediated tumor progression, we performed in vivo rescue experiments combining genetic and lipid supplementation strategies. Wild-type exosomes (LLC^FASN-WT^-EXOs) markedly promoted tumor growth, whereas FFA-deficient exosomes from FASN-KD cells (LLC^FASN-KD^-EXOs) lost this capacity. Strikingly, exogenous FFA supplementation fully restored the protumorigenic effect of FASN-KD exosomes, identifying FFAs as the critical cargo. Furthermore, Rab34 silencing in neutrophils abolished the tumor-promoting effect of wild-type exosomes, a defect partially rescued by exogenous FFAs (Fig. 7A to C). Consistently, intratumoral NETs formation, as measured by MPO–DNA levels, correlated with tumor growth across all groups, with the highest levels in FASN-WT and FFA-rescued groups and marked reduction upon Rab34 silencing (Fig. 7D). These findings establish a mechanistic axis: Cancer cell-derived exosomal FFAs, internalized via Rab34-dependent uptake, reprogram neutrophils to fuel lung cancer progression (Fig. 7E). This work positions exosomal lipid transfer as a key driver of tumor–immune crosstalk and highlights Rab34 and FAO as potential therapeutic targets.

## Discussion

Recently, numerous articles have shown that tumor infiltrating neutrophils are educated in the TME. Importantly, educated neutrophils maintain a cancer-promoting phenotype, which exhibit longer lifespan and release more protumorigenic substances such as MMP-9 and vascular endothelial growth factor to fuel tumor progression [8,10]. Others reported that tumor reprograms neutrophil through lipid accumulation in infiltrated neutrophils [9]. However, little is known about the resource of the lipids accumulated in neutrophils. This study reveals that lung cancer cells transported fatty acids to neutrophils via fatty acid-rich exosomes and enhanced FAO activity and promoted NETs formation to exacerbate lung cancer growth. Additionally, Rab34 was identified as a key regulator for exosome uptake in neutrophils (Fig. 7E).

Within the TME, a dynamic crosstalk occurs between immune cells and tumor cells, mediated by cytokines and other factors, which shapes their mutual behavior [19–21]. We and others have reported that lung cancer-educated neutrophils released NETs to promote tumor growth and metastasis in lung cancer tissues [6,10,33]. However, it is not clear which factor released by lung cancer cells is the key factors to activate neutrophils, thus inducing NETs formation. RNA-seq revealed that lung cancer-educated neutrophils significantly up-regulate lipid metabolism-related genes—a finding validated by enhanced neutral lipid staining (Nile Red/BODIPY 493/503) in tumor-infiltrating neutrophils. We further demonstrated that LDEs alone were sufficient to increase neutral lipid content in neutrophils to a level comparable to full tumor-CM. However, this effect was not observed with exosome-free lung cancer cell-CM. To identify the specific lipids responsible for inducing lipid accumulation and NET formation in neutrophils, we performed HPLC-MS/MS-based lipidomic profiling. The analysis revealed a marked increase in FFA levels in exosome-stimulated neutrophils. The BODIPYTM FL C16 fatty acid trafficking assay showed that lung cancer cells could transport fatty acids to neutrophils by exosomes directly. Additionally, cancer cell-derived exosomes enriched FFAs compared with lung epithelial noncancerous cells. High level of lipids in lung cancer cell-derived exosomes induced lipid accumulation in neutrophils, resulting in NETs formation, when compared with exosomes from noncancerous cells.

FFAs accumulation has been demonstrated to induce hyperactivation of tumor-infiltrating neutrophils [9]. In this process, we found that fatty acid enhanced the CPT1a-promoting FAO pathway, facilitating mtROS production and consequently promoting NETs formation. The protumor effect of exosome-primed neutrophils was abolished by DNase I-mediated NET depletion in vivo, demonstrating that NETs formation is critically involved in neutrophil-mediated tumor promotion. Previous reports found that tumor cells can secret several soluble cytokines to induce NETs formation directly, like interleukin-8, high-mobility group box 1, and granulocyte colony-stimulating factor in vivo and in vitro [34–36]. However, our work showed that exosome-free tumor cell-CM might contain soluble protein above and does not influence lipid levels in neutrophils, further indicating that the cytokines of tumor cell secretion induce NETs formation without promoting synthesis of FFAs and catabolism. Importantly, these also proved that neutrophils educated by tumor cells deliver lipids through exosomes without affecting lipid synthesis. At first glance, the coexistence of increased lipid accumulation and enhanced FAO may appear paradoxical. However, this is resolved by considering lipid flux: The massive influx of exogenous FFAs from LDEs exceeds oxidative capacity, resulting in net lipid accumulation despite FAO up-regulation. Our time-course data show that lipid accumulation precedes FAO activation, and etomoxir inhibits NETs but does not prevent initial lipid accumulation. Thus, FAO is a consequence of lipid overload, linking lipid accumulation to NETs formation. Our findings, integrated with existing evidence, reveal a sophisticated signaling network through which tumor cells educate neutrophils and instruct them to form NETs.

Excepting lipid metabolism changing in tumor-educated neutrophils showed by RNA-seq, the data strongly indicated that Rab34 was significantly up-regulated and negatively correlated with patient prognosis, which was confirmed at the protein expression level. It is well documented that the interaction between GTPase dynamin and Rab family proteins orchestrates key cellular processes, including internalization and the amplification of inflammatory signals [30–32]. Upon silencing GTPase proteins, we identified Rab34 as the primary regulator responsible for the internalization of lung cancer cell-derived exosomes and lung cancer progression induced by NETs. The established interplay between GTPase dynamin and the Rab family illustrates a complex regulatory network that governs cellular processes such as internalization. Silencing GTPase proteins highlights Rab34 as a crucial regulator and underscores its possible function in facilitating the internalization of lipid-rich exosomes within the TME. We further investigated whether Rab34 up-regulation is directly driven by exosomal FFAs. Our preliminary data showed that direct FFA supplementation alone was insufficient to induce Rab34 expression in neutrophils, suggesting that other exosomal components, such as proteins or nucleic acids, may be responsible for its transcriptional regulation. A comprehensive dissection of the upstream signals controlling Rab34 expression represents an important direction for future studies. This internalization process is crucial, as it can facilitate the transfer of mediators of lung cancer cells and promote tumor progression. Targeting Rab34 or its associated pathways may offer a strategic approach to mitigate inflammation and potentially enhance the efficacy of current cancer treatments. Therapeutically targeting this pathway holds translational potential for lung cancer by interrupting a critical communication axis that reprograms neutrophils toward a protumor phenotype.

The translation of these findings into clinical practice involves a multistage pathway. First, therapeutic agents that target Rab34 or specific NET components, such as peptidyl arginine deiminase 4 inhibitors, must be developed and tested for specificity and safety to avoid impairing beneficial immune functions. Second, their efficacy should be validated in advanced preclinical models, including patient-derived xenografts and orthotopic lung models, to confirm the mechanism within a human-relevant TME. Crucially, these novel agents ought to be evaluated in combination with standard therapies like immunotherapy, since NET disruption may help overcome treatment resistance. Concurrently, the development of biomarkers—such as NET signatures detected in liquid biopsies—is essential for identifying patients most likely to respond in future clinical trials, thereby enabling a targeted therapeutic strategy. This approach seeks to convert the protumoral neutrophil niche from a driver of progression into a therapeutic vulnerability.

While our study provides important insights into how LDEs reprogram neutrophils to promote tumor progression, several limitations should be considered. First, the translational potential of targeting Rab34 or NETs in clinical settings requires further validation using patient-derived samples or orthotopic models. Second, although lipidomics identified FFAs as key mediators, the study did not fully characterize the heterogeneity of exosomal lipids or rule out potential contributions from other cargo components. Third, while Rab34 knockdown attenuated exosome uptake and NETs formation, its broader roles in neutrophil function and potential off-target effects were not explored. Additionally, the direct causal relationship between exosomal FFAs, NETs, and clinical outcomes remains associative. Fourth, while our study establishes a critical role for Rab34 in neutrophil-mediated tumor progression, direct validation of Rab34 expression and its correlation with NETs markers in human lung cancer tissues remains to be addressed. Access to clinical samples and patient-derived neutrophils will be essential in future studies to strengthen the translational relevance of our findings. Future studies should measure NETs markers in patient samples to strengthen clinical relevance. Addressing these limitations will be crucial for developing targeted therapies against exosome-mediated neutrophil reprogramming in lung cancer.

Our findings elucidate a crucial tumor-neutrophil interaction, demonstrating the pivotal role of LDEs and the FAO pathway in driving NETs formation and tumor progression. Additionally, the identification of Rab34 as a key regulator of lung cancer cell-derived exosomes uptake and NETs formation suggests that Rab34 may serve as a potential therapeutic target for lung cancer treatment. This study provides a comprehensive understanding of the molecular mechanisms, underlying the interaction between lung cancer cells and neutrophils, and highlights the potential of targeting this interaction for the development of novel therapeutic strategies in the treatment of lung cancer.

## Methods

### Study design

This study investigated the mechanism through which LDEs promote neutrophil reprogramming and tumor progression by inducing NETs formation, employing integrated in vitro and in vivo approaches. RNA-seq of neutrophils treated with lung cancer cell-CM identified altered signaling pathways. Lipidomic profiling via HPLC-MS/MS further characterized lipid accumulation in neutrophils. Functional assays—including exosome uptake, fatty acid transfer, and NETs formation—evaluated metabolic reprogramming and activation of neutrophils. Syngeneic mouse models, using C57BL/6J mice injected with LLC cells, assessed the roles of Rab34 and NETs in vivo. The sample sizes for all animal experiments (*n* = 4 to 5 per group) were justified by prior data to ensure 80% power at an alpha of 0.05, with mice randomized before the initiation of treatment. Exact values of *n* can be found in the figure legends. Herein, the term “*n*” connotes biologically independent samples: specifically, the number of individual animals for in vivo studies and the count of independent cultures or isolations for in vitro analyses. Moreover, every major finding was corroborated in a minimum of 3 separate experiments, and technical replicates were employed solely in instances that are clearly indicated.

### Cell culture

The following cell lines were used: LLC (CL-0140; Procell), A549 (CL-0016; Procell), and HL-60 (CL-0110; Procell), cultured in Dulbecco’s modified Eagle’s medium, F12, and RPMI 1640 media (KeyGEN, China), respectively; Beas2B (FH0319; Fuheng) and MLE (FH1103; Fuheng) cells, both cultured in RPMI 1640. All cultures were maintained at 37 °C with 5% CO₂ in medium containing 10% fetal bovine serum (Sunrise, USA) and 1% penicillin–streptomycin (Beyotime, China).

### Neutrophil collection

Human neutrophil-like cells were modeled by differentiating HL-60 cells into dHL-60 cells using an established protocol. In brief, cells were treated with 1.3% dimethyl sulfoxide in complete RPMI 1640 medium (supplemented with 10% fetal bovine serum) for 5 to 7 d to induce granulocytic differentiation. The mouse BMN isolation was carried out according to the protocol as described by our previously papers [10]. Tumor-infiltrated neutrophils were isolated by BD FACSAria III from tumor tissue single-cell suspension.

### Lentiviral production

Pseudoviral particles for gene silencing were generated using control short hairpin (sh)RNA plasmids and shRNA plasmids specific for human Rab34, human and mouse FASN, human SMPD2, and human CPT1a, which were obtained from the MiaoLing Plasmid Platform (Wuhan, China). These plasmids were cotransfected with the helper plasmids pMD2.G and psPAX2 into 293T packaging cells. The viral supernatant was collected 48 h after transfection and used to transduce the corresponding cells for gene suppression. After transduction, the cells underwent puromycin selection for 3 d. Finally, western blot analysis confirmed the knockdown efficiency of the target proteins.

### Isolation of LDEs

Lung cancer cell-CM was prepared as described. Exosomes were isolated via ultracentrifugation following an established protocol [37]. Briefly, the medium underwent serial centrifugation steps at 4 °C: 350 g and 2,000 g for 10 min each, followed by 12,000 g for 40 min, to remove cells and debris. The supernatant was then passed through a 0.2-μm filter (PN4612; Pall, Westborough, MA, USA) and subjected to ultracentrifugation (Beckman Coulter, Brea, CA, USA) at 120,000 g for 70 min (4 °C) to pellet the exosomes.

The condensed pellet was washed with ice-cold phosphate-buffered saline (PBS) and centrifuged again at 120,000 g for another 70 min at 4 °C. The resulting pelleted exosomes were suspended in PBS, and concentrations of exosome proteins were measured using the BCA Protein Assay Kit (Beyotime, Shanghai, China).

Isolated exosomes were analyzed for particle size and zeta potential on a Malvern ZetaSizer Nano system. Furthermore, western blotting was performed to confirm the presence of the exosomal marker CD9 and the absence of the endoplasmic reticulum protein calnexin, serving as a negative control. The morphology of the exosomes was observed using a biological electron microscope.

### GFP-positive exosomes preparation

To produce CD9-GFP pseudoviruses, 293T cells were cotransfected with the pLV3-CMV-CD9-EGFP-Puro plasmid (Miaoling Biology, China) and the packaging plasmids pMD2.G and psPAX2. The supernatant containing the viral particles was harvested after 48 h and employed to infect LLC cells. Transfection efficiency was analyzed by flow cytometry after 3 consecutive days of puromycin screening. Exosomes from CD9-GFP-positive were isolated as above, and GFP intensity of exosomes was confirmed by a Cytation 3 Cell Imaging MultiMode Reader.

### RNA-seq for tumor-educated neutrophils

CM was collected from LLC and A549 cells after 48-h culture in serum-free Dulbecco’s modified Eagle’s medium and F12 medium, respectively. Cell-free medium, processed identically in the absence of cells, served as the control. Neutrophils were then stimulated with or without CM for 4 h at 37 °C, after which cell pellets were harvested. Total RNA was extracted using TRIzol reagent (Invitrogen). RNA-seq was performed by CapitalBio Technology (Beijing), and the resulting raw reads were quality-controlled with FastQC, adapter-trimmed, and aligned to the reference genome using HISAT2. Gene expression was quantified by featureCounts, and differentially expressed genes were identified with DESeq2 (∣log₂FC∣ > 1, adjusted *P* < 0.05). Functional enrichment of differentially expressed genes was analyzed via Reactome and Kyoto Encyclopedia of Genes and Genomes pathway databases.

### Nile Red staining

Neutrophils were treated with lung cancer cell-CM or 40 μg/ml exosomes for 0 to 4 h at 37 °C. Cells were then stained with 0.5 μM Nile Red for 10 min at room temperature, followed by 3 washes with PBS. Fluorescence intensity was measured using either a Cytation 3 Cell Imaging Multi-Mode Reader (BioTek; excitation/emission: 559/635 nm) or a BD FACSCanto II flow cytometer.

### Mitochondrial mass

Neutrophils (1 × 10^7^/ml) were incubated with 40 μg/ml exosomes for 4 h at 37 °C. After being washed 3 times with PBS, the cells were stained with 70 nM MitoTracker for 10 min at room temperature and washed again. Fluorescence intensity was then measured using a Cytation 3 Cell Imaging Multi-Mode Reader (BioTek, Winooski, VT).

### Mitochondrial ROS

Neutrophils (1 × 10^7^/ml) were treated with 40 μg/ml exosomes for 4 h at 37 °C. After washing 3 times with PBS, the cells were incubated with 1 μM MitoSOX for 10 min at room temperature. Following another wash cycle, fluorescence intensity was measured using a Cytation 3 Cell Imaging Multi-Mode Reader (BioTek).

### Confocal microscopy

Neutrophils were first incubated with lung cancer cell-CM for 4 h at 37 °C. The cells were then allowed to adhere onto poly-L-lysine-coated glass coverslips in culture plates. After fixation, the adherent neutrophils were stained with BODIPY 493/503 (for neutral lipids) and Hoechst (for nuclei) and finally imaged using a Zeiss LSM880 confocal microscope (Oberkochen, Germany).

For exosome uptake, mouse BMNs were incubated with GFP-exosomes for 2 h and then allowed to adhere to glass coverslips for 30 min. Following fixation, the adherent neutrophils were stained with Dil to label the cell membrane and with Hoechst for 10 min. Images were acquired using a Zeiss LSM880 confocal microscope (Oberkochen, Germany).

### Enzyme-linked immunosorbent assay

Following a 4-h stimulation with lung cancer cell-CM at 37 °C, neutrophil supernatants were collected by centrifugation (500 g, 5 min, 4 °C). MPO–DNA levels in the supernatant were measured using a specific enzyme-linked immunosorbent assay kit (Jiangsu Meimian) according to the manufacturer’s protocol.

To quantify intratumoral MPO–DNA concentrations, equal weights of tumor tissue were homogenized in an equivalent volume of PBS. The homogenate was centrifuged, and the supernatant was collected. MPO–DNA levels were then measured using enzyme-linked immunosorbent assay kits.

### Fatty acid detection assay

After a 4-h incubation with LDEs at 37 °C, neutrophils were collected by centrifugation at 500 g for 5 min at 4 °C. The FFA content in both neutrophils and exosomes was then quantified using a commercial FFA assay kit (Enzyme-linked Biotechnology Co., Ltd, China), in accordance with the manufacturer’s instructions.

### Fatty acid transfer assay

LLC or A549 cells were first labeled by culturing with BODIPY FL C16—a fluorescent fatty acid analog—for 24 h. After washing with PBS, the labeled cells were further incubated in serum-free RPMI 1640 medium for 48 h. The CM was collected, and exosomes were isolated via ultracentrifugation as previously described. Subsequently, BMNs or dHL-60 cells were incubated with these labeled exosomes for 4 h. Fluorescence intensity (Ex/Em: 505/512 nm) in tumor cells, exosomes, and neutrophils was measured using a Cytation 3 Cell Imaging Multi-Mode Reader.

### Western blot

Isolated LDEs were lysed in sodium dodecyl sulfate-polyacrylamide gel electrophoresis loading buffer (P0015F, Beyotime) and denatured at 100 °C for 10 min. Proteins were separated on 12% sodium dodecyl sulfate-polyacrylamide gels and transferred to membranes. The membranes were probed with the following primary antibodies (1:1,000 dilution): anti-CitH3 (ab5103, Abcam), anti-Rab34, anti-CPT1a, anti-CD9, anti-CD63, and anti-actin (all from Cell Signaling Technology). Immunoreactive bands were visualized using an enhanced chemiluminescence chemiluminescent detection system (Thermo Scientific) and imaged with a Tanon imaging system. Band intensity was quantified using ImageJ software (National Institutes of Health).

### Fatty acid mixture

The fatty acid mixture was prepared according to a published protocol [24]. Briefly, fatty acid stocks (200 mM) of palmitic (C16:0), stearic (C18:0) and oleic acid (C18:1) were prepared in 96% ethanol by heating at 70 °C with shaking, then equilibrated at 37 °C. Each stock was diluted 1:10 in prewarmed 10% FA-free bovine serum albumin (BSA) to a final concentration of 20 mM. The mixtures were vortexed (30 s) and incubated with gentle agitation at 55 °C (15 min) followed by 37 °C. The solutions were then filtered through a 0.2-μm membrane. Neutrophils were treated with each fatty acid at a final concentration of 100 μM for 4 h. Control cells received equivalent BSA and ethanol vehicle.

### Quantitative real-time PCR assay

BMNs were incubated with LLC-EXOs (40 μg/ml, 4 h) and subjected to RNA extraction using TRIzol. After cDNA synthesis (HiScript RT-PCR Kit), quantitative PCR was carried out on an ABI PRISM 7500 system (95 °C for 10 min; 40 cycles of 95 °C/10 s and 60 °C/34 s). Gene expression of FAO/oxidative phosphorylation-related targets (e.g., cpt1a, cpt2, acadvl, and ndufs1) was analyzed with β-actin as reference (primers in Table S1).

### cfDNA detection

The concentration of cfDNA in the supernatant was quantified using Sytox Green Nucleic Acid Staining (Invitrogen). Briefly, 200 μl of supernatant was mixed with 1 μM Sytox Green in a 96-well plate and incubated in the dark at room temperature for 20 min. Fluorescence intensity was then measured at an emission wavelength of 523 nm using a Cytation 3 Cell Imaging Multi-Mode Reader (BioTek).

### Lipidomics analysis

For neutrophils lipidomics analysis, BMNs (1 × 10^7^/ml) were stimulated by LLC-exosomes (40 μg/ml) for 4 h at 37 °C. The neutrophil suspension was centrifuged, and the neutrophils were collected and quantified by HPLC-MS/MS analysis as described previously [38]. All identified lipid species were verified using authentic standards. Metabolite abundances were normalized to neutrophil counts, and integrated peak areas (reflecting metabolite concentrations) were quantified with MetaboAnalyst 3.0 [39].

### Seahorse extracellular flux analysis

To measure fatty acid oxidation, BMNs were seeded at 200,000 cells per well in Seahorse XF96 plates with or without exosomes (40 μg/ml) and centrifuged at 400 g for 5 min to settle the cells. After 4 h, OCRs were determined using a Seahorse XFe96 analyzer (Agilent, CA, USA) according to the manufacturer’s protocol. One hour before the assay, the medium was replaced with FAO assay medium, consisting of Krebs–Henseleit buffer supplemented with 2.5 mM glucose, 0.5 mM carnitine, and 5 mM Hepes (pH 7.4). Palmitate was added to a final concentration of 0.1 mM immediately prior to measurement. The following compounds were injected sequentially during the OCR run at these final concentrations: 40 μM etomoxir, 1.5 μM oligomycin, 1 μM carbonyl cyanide *p*-trifluoromethoxyphenylhydrazone, and 0.5 μM each of rotenone and antimycin A.

### Evaluation of cell viability

Neutrophils (BMNs or dHL-60) were treated with or without LDEs (40 μg/ml) for 4 h and in the presence or absence of DNase I (100 U/ml). The CM was subsequently collected, centrifuged to remove debris, and transferred to LLC or A549 cells in 96-well plates. Following incubation for 24, 48, or 72 h, we assessed cell viability using the Cell Counting Kit-8 assay according to the manufacturer’s instructions. Absorbance was measured at 450 nm using a microplate reader.

### Preparation of Rab34-siRNA liposomes

Liposomes were prepared as previously described [38] by combining L-α-phosphatidylcholine, cholesterol, PEG2000-DSPE, and siRNA (Rab34 or control) at a molar ratio of 70:30:2:2.8 in chloroform. The solvent was evaporated using a rotary vacuum evaporator, and the resulting lipid film was hydrated with 250 mM ammonium sulfate via water bath sonication for 5 min. The liposomes were then resuspended in PBS (137 mM NaCl, 2.7 mM KCl, 8 mM Na₂HPO₄, and 2 mM KH₂PO₄, pH 7.4) to form micellar colloids. The Rab34-siRNA sequences used are provided in Table S2.

### Mouse models

Wild-type male C57BL/6J mice (6 to 8 weeks old) were sourced from Xuzhou Medical University Animal Center and maintained under standard conditions. All animal experiments were performed in compliance with the university’s ethical guidelines following approval by the Institutional Animal Ethics Committee.

For neutrophils and LDEs coinjection mouse model, mice were inoculated with 3 × 10^5^/100 μl of LLC cells subcutaneously according to our previously report [10]. When tumor volume reached to 50 mm^3^, 5 × 10^5^ neutrophils or neutrophils precultured with 40 μg/ml LDEs for 2 h were injected on the paratumor side every 3 d for 3 times. Tumor growth was monitored every other day.

For NETs depletion in vivo, mouse lung cancer model was established as above. DNase I (5 mg/kg) was injected intraperitoneally every day after tumor volume reaching to 50 mm^3^. Tumor growth was monitored every other day.

For Rab34 knockdown in neutrophils, mouse lung cancer model was established as above. BMNs were cultured with Rab34-siRNA liposomes for 4 h and injected into the paratumor side after washing with PBS every 3 d for 3 times. Mice were monitored every other day for tumor development. Upon completion of the 20-d treatment period, the animals were euthanized and the tumors were collected for analysis.

To establish the orthotopic lung cancer model, 8 × 10^5^ LLC cells suspended in 50 μl of Matrigel were implanted into the left lung of each mouse. One week postimplantation, the subjects were allocated into 3 distinct experimental groups: a PBS control, a group receiving BMNs treated with control siRNA and LLC-derived exosomes (BMNs-Ctrl-siRNA+EXOs), and a group receiving BMNs treated with Rab34 siRNA and LLC-derived exosomes (BMNs-Rab34-siRNA+EXOs). These interventions were administered via retro-orbital injection every 3 d for a total of 3 doses. Throughout the study, body weights were recorded. After 20 d, all animals were humanely euthanized, and the tumor tissues were harvested for subsequent analysis.

### Isolation of tumor-infiltrating immune cells

Tumor tissues were harvested, minced, and mechanically dissociated. The single-cell suspensions were filtered through a 40-μm cell strainer. Cells were stained with fluorochrome-conjugated antibodies specific for CD45, CD11b, Ly6G, and F4/80 (Biolegend). Using a BD FACSAria III flow cytometer, we sorted tumor-infiltrating neutrophils (CD45^+^/CD11b^+^/Ly6G^+^) and macrophages (CD45^+^/CD11b^+^/F4/80^+^). The sorted populations were collected directly into RIPA lysis buffer for protein extraction, and Rab34 expression was subsequently assessed by western blot.

### Immunofluorescent staining

Tumor tissues were fixed in 4% PFA, OCT-embedded, and cryo-sectioned (5 μm). After permeabilization (0.1% Triton X-100) and blocking (10% BSA), sections were stained with anti-CitH3/MPO antibodies overnight, followed by Alexa Fluor-conjugated secondary antibodies. Images were acquired on Leica DM4000B and TCS SP8 systems.

### Statistical analysis

Unless otherwise specified, data are presented as means ± standard error of the mean (SEM). Statistical significance was determined using GraphPad Prism 8.0, with unpaired *t* tests for 2-group comparisons and 1-way ANOVA (Tukey’s test) for multiple groups. A *P* value < 0.05 was deemed significant.

## Data Availability

The data of this study are available from the corresponding author upon reasonable request.
