# Supplementary material for: Tumor-Derived Exosomal Fatty Acids Reprogram Neutrophils to Drive Neutrophil Extracellular Traps Formation and Lung Cancer Progression
Source: Research (Wash D C). 2026 May 11;9:1278. doi: 10.34133/research.1278 (PMC13158458; doi:10.34133/research.1278)
Supplement: Supplementary 1 — Figs. S1 to S7 Tables S1 and S2 [file research.1278.f1.pdf]

Tumor-derived Exosomal Fatty Acids Reprogram Neutrophils to Drive Neutrophil Extracellular Traps Formation and Lung Cancer Progression

Authors: Lulu Han[1,2,3#*], Yuxin Chen[1,2,3#], Ruchen Wu[1], Junze Chen[1], Zhaokai Chen[1], Ying Cai[1], Shuxin Huang[1], Haoqing Gu[1], Xiaoyi Zhuang[1], Yanfang Lv[1], Huizhong Li[1,2,3], Liantao Li[2,3*], Gang Wang[1,2,3*]

Affiliations:

[1]Cancer Institute, Xuzhou Medical University, 209 Tongshan Road, Xuzhou 221004, China.

[2]Center of Clinical Oncology, The Affiliated Hospital of Xuzhou Medical University, 99 West Huaihai Road, Xuzhou 221002, China.

[3]Jiangsu Center for the Collaboration and Innovation of Cancer Biotherapy, Xuzhou Medical University, 209 Tongshan Road, Xuzhou 221004, China.

[#]Lulu Han and Yuxin Chen contributed equally to this work.

*Corresponding author. E-mail: LLHan@xzhmu.edu.cn (Lulu Han). Email: liliantao@xzhmu.edu.cn. (Liantao Li). E-mail: wangg@xzhmu.edu.cn. (Gang Wang)

Supplementary Fig. 1 – 7

Supplementary tables 1 – 2

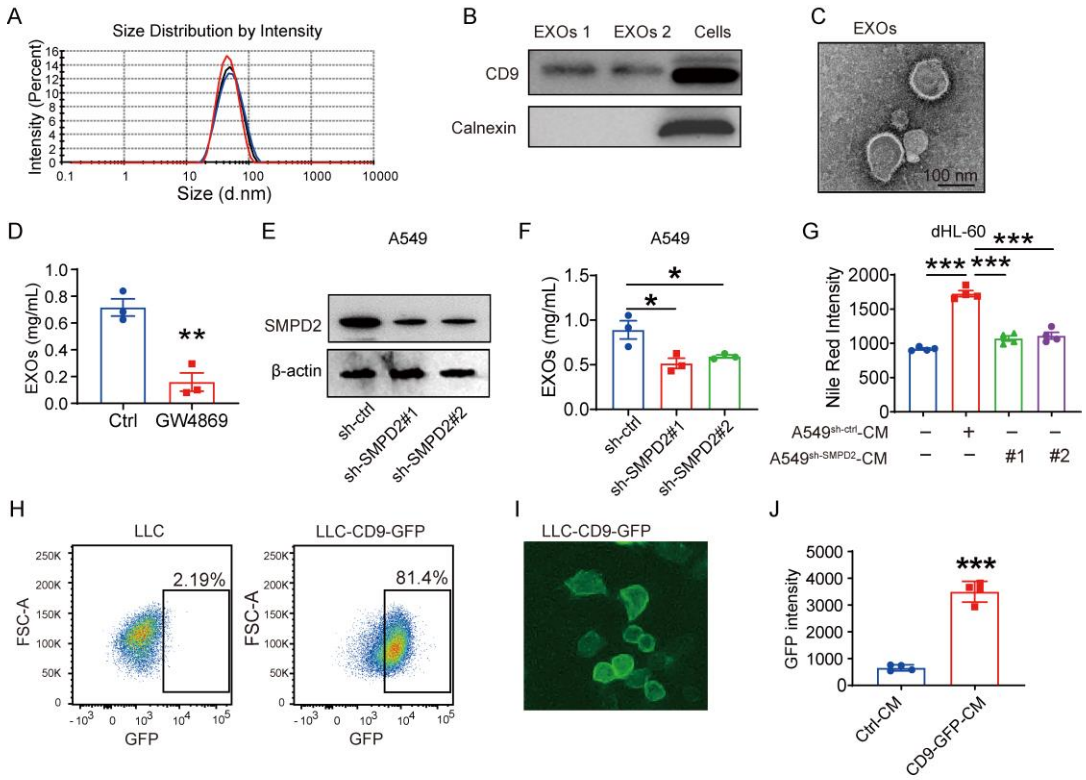

Supplementary Fig. 1. Lung cancer cell-derived exosomes exacerbated the lipid accumulation of neutrophils and their pro-tumoral function. (A) Size intensity curves and the mean size of exosomes were measured by Malvern. **(B)** Exosome-positive marker, CD9, and exosome-negative marker, Calnexin, were measured by western blot. **(C)** The LLC-derived exosomes were visualized using transmission electron microscopy. **(D)** Quantitative analysis of exosomes in the supernatant of A549 cells treated with DMSO (Ctrl) or 10 μM GW4869 for 48 hours. **(E)** SMPD2 expression was assessed by western blot in A549 cells transduced with the indicated shRNAs. (F) Quantitative analysis of exosomes in the supernatant of A549 cells. **(G)** Human dHL-60 neutrophils were cultured in the indicated CM. Lipid levels were subsequently quantified via Nile Red staining. **(H)** The ratio of GFP in LLC cells was measured by flow cytometry. **(I)** LLC-CD9-GFP cells representative image taken by a fluorescence microscope. **(J)** GFP intensity was measured by Cytation 3 Cell Reader in LLC-CD9-GFP-CM. Data are presented as the mean ± SEM, (n=3 - 4, $*P < 0.05$, $**P < 0.01$, $***P < 0.001$).

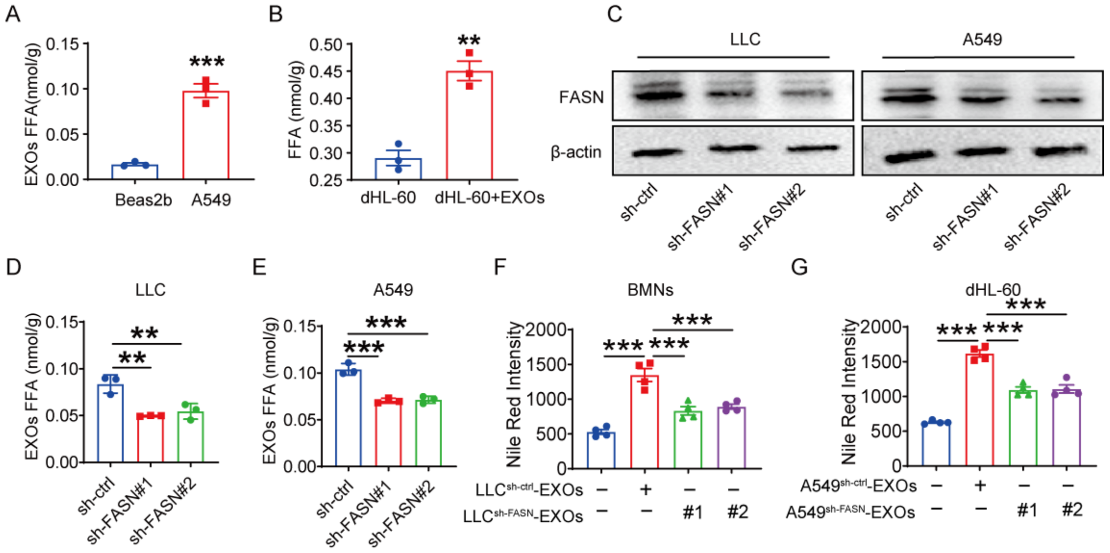

Supplementary Fig. 2. Lung cancer cell-derived exosomes deliver free fatty acids into neutrophils to induce lipid accumulation. (A) FFA levels were measured in exosomes derived from Bease2b and A549 cells. **(B)** FFA levels were measured in dHL-60 cultured with exosomes. **(C)** FASN expression was assessed by western blot in LLC and A549 cells transduced with the indicated shRNAs. **(D)** FFA levels were quantified in exosomes derived from LLC cells. (E) FFA levels were quantified in exosomes derived from A549 cells. **(F)** BMNs were cultured in the indicated exosomes. Lipid levels were subsequently quantified via Nile Red staining. **(G)** Human dHL-60 cells were cultured in the indicated exosomes. Lipid levels were subsequently quantified via Nile Red staining. Data are presented as the mean ± SEM, (n=3 - 4, $**P < 0.01$, $***P < 0.001$).

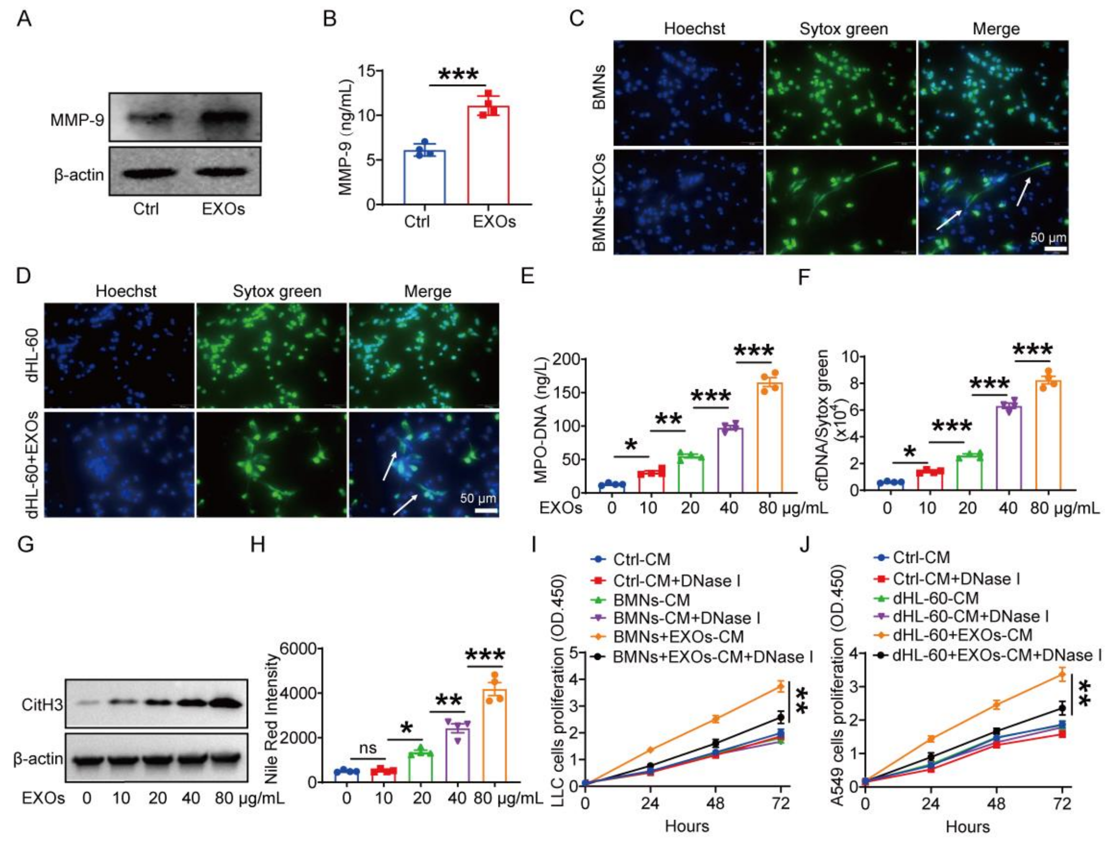

Supplementary Fig. 3. Lung cancer cell-derived exosomes promote neutrophil-induced tumor progression in a NETs-dependent manner. (A) MMP-9 expression in BMNs was assessed via western blot following stimulation with or without LLC-exosomes. **(B)** The levels of MMP-9 in the supernatants were quantified by ELISA under the same stimulation conditions. **(C)** IF imaging visualized NETs released from BMNs following stimulation with LLC-exosomes. **(D)** NETs released from dHL-60 cells stimulated with A549-exosomes were visualized by IF. **(E)** MPO-DNA levels in the supernatants of BMNs stimulated with LLC-exosomes at the indicated concentrations were quantified by ELISA. **(F)** cfDNA levels in the supernatants from BMNs were measured using Sytox Green staining. **(G)** CitH3 expressions were measured by western blot. **(H)** Lipid levels in the BMNs stimulated with exosomes at the indicated concentrations. **(I)** LLC cells were cultured in CM (± DNase I) from BMNs pre-stimulated with LLC-derived exosomes, and proliferation was measured by CCK-8 assay. **(J)** A549 cells were cultured in CM (± DNase I) from dHL-60 cells pre-stimulated with A549-derived exosomes, followed by CCK-8 proliferation assay. Data are presented as the mean ± SEM, (n=4, $^{*}P < 0.05$, $^{**}P < 0.01$, $^{***}P < 0.001$).

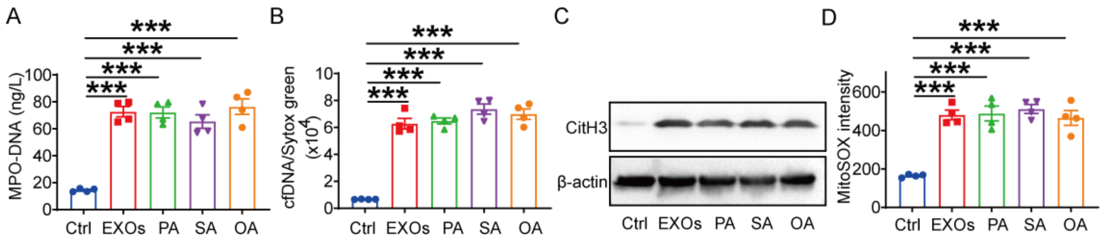

Supplementary Fig. 4. Free fatty acids induce NETosis and mitochondrial ROS production. (A-D) BMNs were incubated for 4 hours with LLC-derived exosomes or with equimolar concentrations (100 μM) of palmitic, stearic, or oleic acid (PA, SA, OA). **(A)** Supernatant MPO-DNA levels were quantified by ELISA. **(B)** cfDNA levels in BMN supernatants were assessed using Sytox Green staining. **(C)** CitH3 expression was analyzed via western blot. **(D)** mtROS production was measured using mitoSOX staining. Data are presented as the mean ± SEM, (n=3 - 4, ****P* < 0.001).

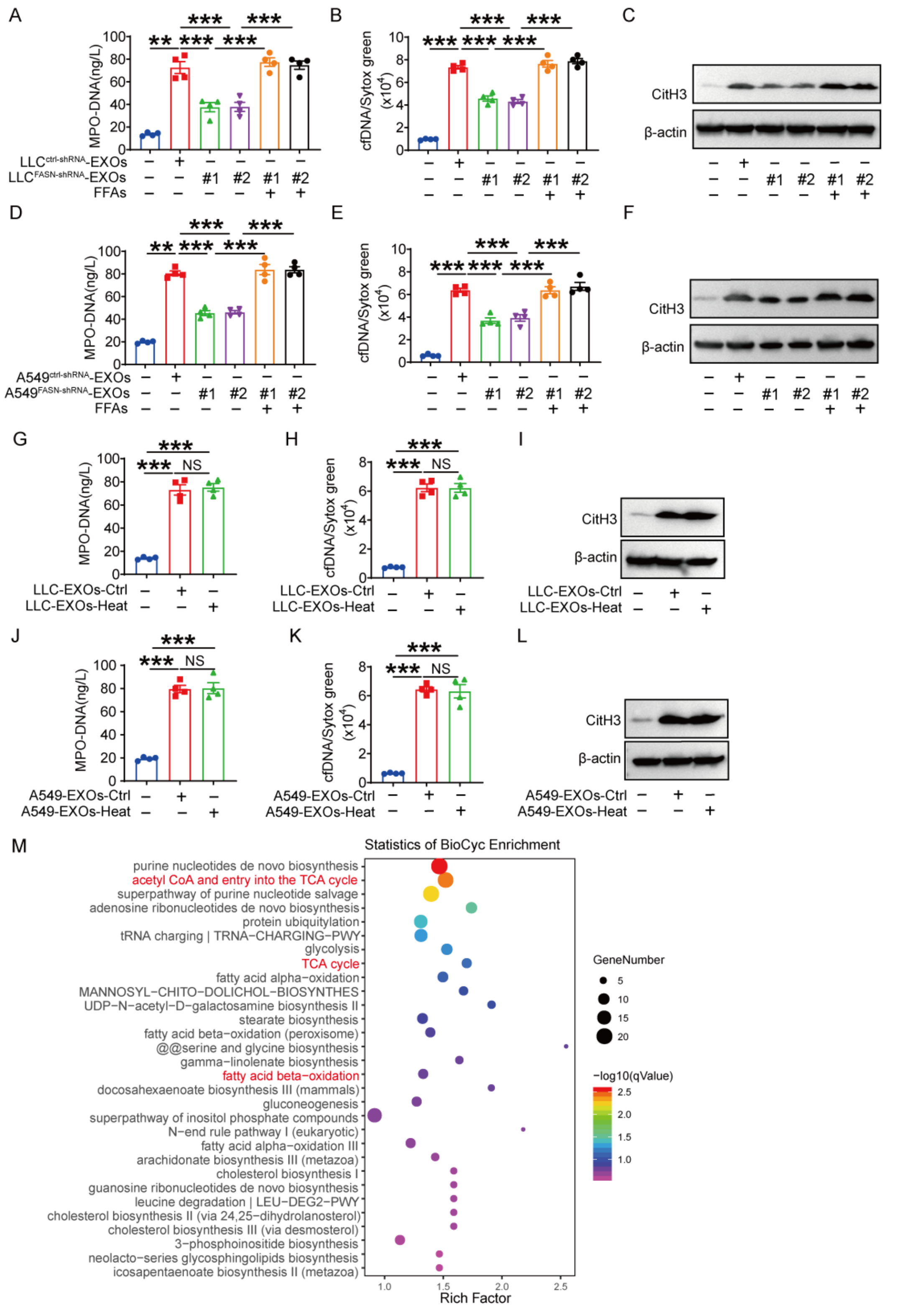

Supplementary Fig. 5. Lung cancer-derived exosomal fatty acids promote NETs formation through FAO pathways activation in neutrophils.

(A-D) BMNs were incubated for 4 hours with exosomes derived from LLC cells with or without FASN knockdown or FFAs. (A) MPO-DNA levels in the supernatant were

quantified by ELISA. (B) Supernatant cfDNA levels were measured using Sytox Green staining. (C) CitH3 expression was evaluated by western blot. (D-F) dHL-60 cells were incubated for 4 hours with exosomes from A549 cells with or without FASN knockdown or FFAs. (D) MPO-DNA in the supernatant were quantified by ELISA (E) Supernatant cfDNA levels were measured using Sytox Green staining. (F) CitH3 expression was analyzed by western blot. (G-I) BMNs were incubated for 4 hours with exosomes derived from LLC cells or with the same exosomes following heat inactivation (90°C, 10 min). (G) MPO-DNA levels in the supernatant were quantified by ELISA. (H) Supernatant cfDNA levels were measured using Sytox Green staining. (I) CitH3 expression was analyzed by western blot. (J-L) dHL-60 cells were incubated for 4 hours with exosomes derived from A549 cells or with the same exosomes following heat inactivation (90°C, 10 min). (J) MPO-DNA levels in the supernatant were quantified by ELISA. (K) Supernatant cfDNA levels were measured using Sytox Green staining. (L) CitH3 expression was analyzed by western blot. (M) Pathways enrichment analysis (Statistics of BioCyc Enrichment) in A549-CM-treated neutrophils compared with control neutrophils. Data are presented as the mean ± SEM, (n=3 - 4, **$P < 0.01$, ***$P < 0.001$, NS: not significant).

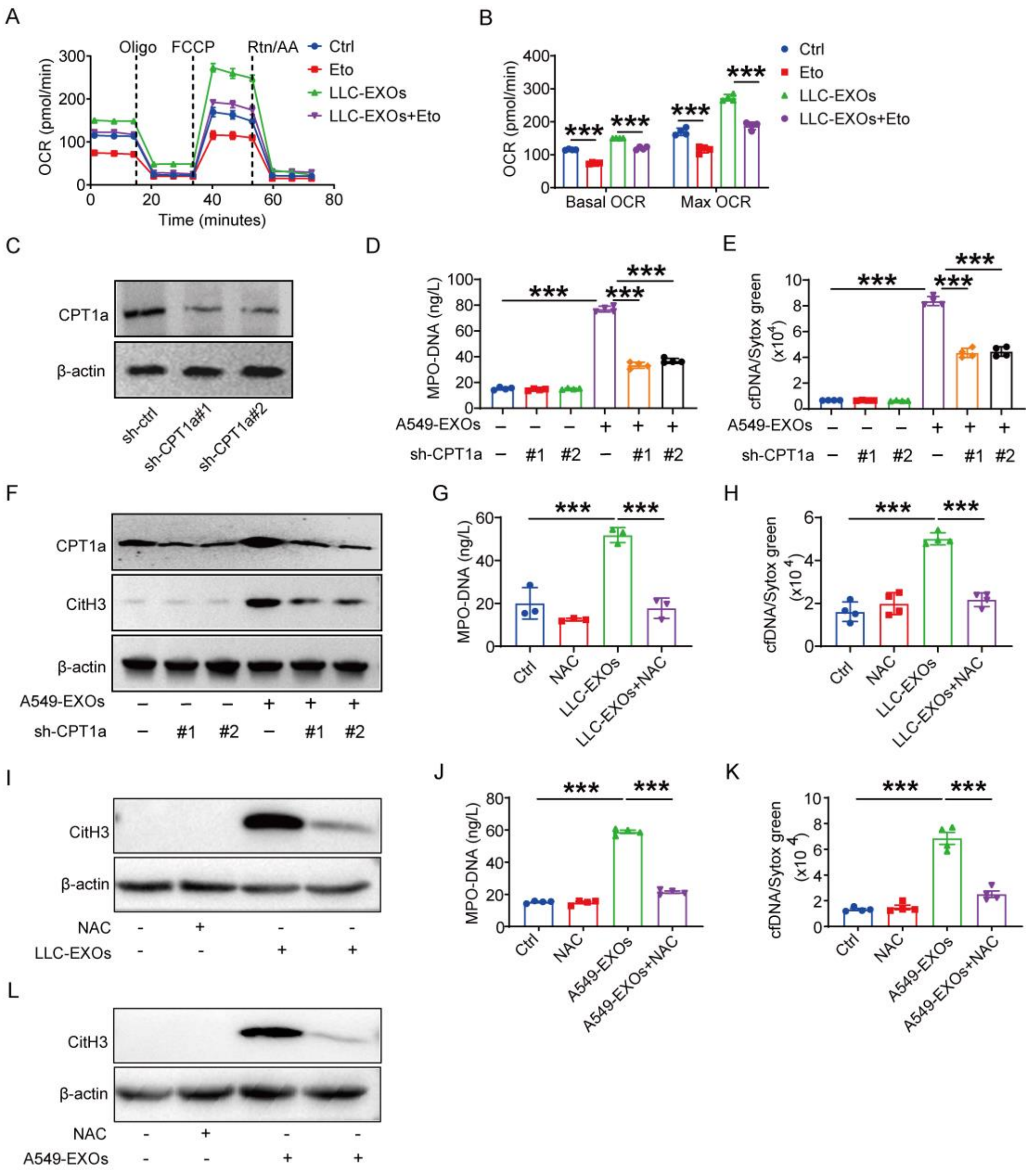

Supplementary Fig. 6. FAO and ROS are required for LDE-induced NETs formation. (A) The oxygen consumption rate (OCR) of BMNs stimulated with LLC-exosomes, with or without etomoxir, was measured using a Seahorse analyzer. **(B)** Both the basal and maximal OCR values were subsequently analyzed. **(C)** CPT1a expression in HL-60 cells transduced with the indicated shRNAs was assessed by western blot. (D-F) Control or CPT1a knockdown dHL-60 cells were cultured for 4 hours in the presence or absence of A549-derived exosomes. **(D)** MPO-DNA levels in the supernatant were quantified by ELISA. **(E)** Supernatant cfDNA levels were measured using Sytox Green staining. **(F)** CitH3 expression was analyzed by western blot. **(G-I)** Mouse BMNs were cultured with LLC-EXOs with 10 μM NAC for 4 hours. **(G)** MPO-DNA levels were

measured by ELISA. **(H)** cfDNA levels were measured by Sytox green staining. **(I)** CitH3 expressions were measured by western blot. **(J-L)** dHL-60 neutrophils were cultured with A549-EXOs with 10 μM NAC for 4 hours. **(J)** MPO-DNA levels were measured by ELISA. **(K)** cfDNA levels were measured by Sytox green staining. **(L)** CitH3 expressions were measured by western blot. Data are presented as the mean ± SEM, (n=3 - 4, ***$P < 0.001$)

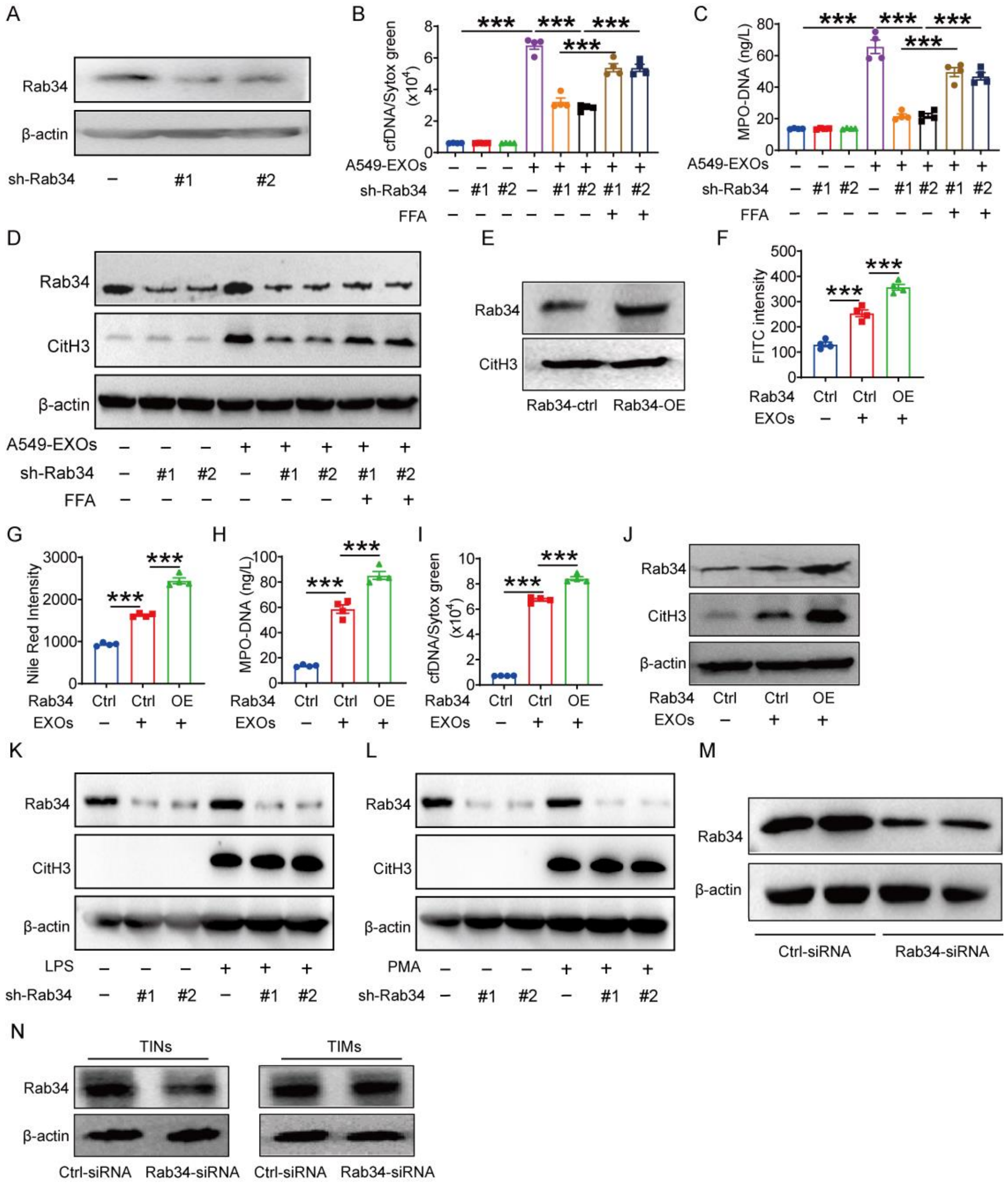

Supplementary Fig. 7. Rab34 promotes NETs formation from neutrophils by facilitating the uptake of lung cancer cell-derived exosomes. (A) Rab34 expression

was knocked down in HL-60 cells using shRNA, as confirmed by western blot analysis. **(B-D)** Control or Rab34-knockdown dHL-60 cells were then cultured for 4 hours with A549-derived exosomes, either in the presence or absence of FFAs. **(B)** MPO-DNA complexes in the supernatant were quantified by ELISA. **(C)** The supernatant levels of cfDNA were measured using Sytox Green staining. **(D)** CitH3 expression was assessed by western blot. **(E)** Rab34 was overexpressed in HL-60 cells, which was confirmed by western blot analysis. **(F-J)** Control or Rab34-overexpressing dHL-60 cells were subsequently incubated for four hours with FITC-labeled exosomes derived from A549 cells. **(F)** FITC levels in control or Rab34-overexpressing dHL-60 cells were measured. **(G)** Lipid levels were assessed via Nile red staining. **(H)** MPO-DNA levels were quantified by ELISA. **(I)** The cfDNA was determined using Sytox green staining. **(J)** CitH3 expression was analyzed by western blot. (**K** and **L**) Human HL-60-differentioned neutrophils with or without Rab34 knockdown were cultured with LPS (K, 1 μg/mL) or PMA (L, 400 nM) for 4 hours. CitH3 expressions were measured by western blot. **(M)** Rab34 was knocked down using siRNA liposomes in BMNs *in vitro*. Rab34 expression was assessed by western blot. **(N)** Western blotting detected Rab34 expression in tumor-infiltrating neutrophils (TINs) and macrophages (TIMs). Data are presented as the mean ± SEM, (n=3 - 4, ***$P < 0.001$).

Supplementary Table 1. Sequences of primer sets used in qRT-PCR

Gene	Sequence (5'-3')
Cpt1a Fwd	5´-TACCACAGAGGCCGTTACTTCA-3´
Cpt1a Rev	5´-AAATAGGTCTGCCGACACTTCG-3´
Cpt1b Fwd	5´-GCACACCAGGCAGTAGCTTT-3´
Cpt1b Rev	5´-CAGGAGTTGATTCCAGACAGGTA-3´
Cpt1c Fwd	5´-TCTTCACTGAGTTCCGATGGG-3´
Cpt1c Rev	5´-ACGCCAGAGATGCCTTTTCC-3´
Cpt2 Fwd	5´-CAGCACAGCATCGTACCCA-3´
Cpt2 Rev	5´-TCCCAATGCCGTTCTCAAAAT-3´
Acox1 Fwd	5´-TAACTTCCTCACTCGAAGCCA-3´
Acox1 Rev	5´-AGTTCCATGACCCATCTCTGTC-3´
Acadm Fwd	5´-AGGGTTTAGTTTTGAGTTGACGG-3´
Acadm Rev	5´-CCCCGCTTTTGTCATATTCCG-3´
Acadl Fwd	5´-TCTTTTCCTCGGAGCATGACA-3´
Acadl Rev	5´-GACCTCTCTACTCACTTCTCCAG-3´
Acadvl-Fwd	5´-CTACTGTGCTTCAGGGACAAC-3´
Acadvl-Rev	5´-CAAAGGACTTCGATTCTGCCC-3´
Sdhb Fwd	5´-AATTTGCCATTTACCGATGGGA-3´
Sdhb Rev	5´-AGCATCCAACACCATAGGTCC-3´
Uqcrc1 Fwd	5´-AGACCCAGGTCAGCATCTTG-3´
Uqcrc1 Rev	5´-GCCGATTCTTTGTTCCCTTGA-3´
Cox4i1 Fwd	5´-ATTGGCAAGAGAGCCATTTCTAC-3´
Cox4i1 Rev	5´-CACGCCGATCAGCGTAAGT-3´
Atp5j Fwd	5´-TATTGGCCCAGAGTATCAGCA-3´
Atp5j Rev	5´-GGGGTTTGTCGATGACTTCAAAT-3´
β-Actin Fwd	5´-GGCTGTATTCCCCTCCATCG-3´
β-Actin Rev	5´-CCAGTTGGTAACAATGCCATGT-3´

Supplementary Table 2. Sequences of Rab34-siRNA

Gene	Sequence (5'-3')
Rab34-siRNA1 F	5´- GGAAGACCUGUCUCAUUAATT-3´
Rab34-siRNA1 R	5´-UUAAUGAGACAGGUCUUCCTT -3´
Rab34-siRNA2 F	5´- GGAAAGGUUCAAGUGCAUUTT-3´
Rab34-siRNA2 R	5´-AAUGCACUUGAACCUUUCCTT -3´
